# Eocene Shark Teeth From Peninsular Antarctica: Windows to Habitat Use and Paleoceanography

**DOI:** 10.1029/2024PA004965

**Published:** 2024-11-05

**Authors:** Gabriele Larocca Conte, Adam Aleksinski, Ashley Liao, Jürgen Kriwet, Thomas Mörs, Robin B. Trayler, Linda C. Ivany, Matthew Huber, Sora L. Kim

**Affiliations:** 1 Life & Environmental Sciences, University of California Merced, Merced, CA, USA; 2 Earth, Atmospheric, and Planetary Sciences, Purdue University, West Lafayette, IN, USA; 3 Department of Paleontology, University of Vienna, Vienna, Austria; 4 Department of Paleobiology, Swedish Museum of Natural History, Stockholm, Sweden; 5 Bolin Centre for Climate Research, Stockholm University, Stockholm, Sweden; 6 Earth and Environmental Sciences, Syracuse University, Syracuse, NY, USA

## Abstract

Eocene climate cooling, driven by the falling *p*CO_2_ and tectonic changes in the Southern Ocean, impacted marine ecosystems. Sharks in high-latitude oceans, sensitive to these changes, offer insights into both environmental shifts and biological responses, yet few paleoecological studies exist. The Middle-to-Late Eocene units on Seymour Island, Antarctica, provide a rich, diverse fossil record, including sharks. We analyzed the oxygen isotope composition of phosphate from shark tooth bioapatite (δ^18^O_p_) and compared our results to co-occurring bivalves and predictions from an isotope-enabled global climate model to investigate habitat use and environmental conditions. Bulk δ^18^O_p_ values (mean 22.0 ± 1.3%o) show no significant changes through the Eocene. Furthermore, the variation in bulk δ^18^O_p_ values often exceeds that in simulated seasonal and regional values. Pelagic and benthic sharks exhibit similar δ^18^O_p_ values across units but are offset relative to bivalve and modeled values. Some taxa suggest movements into warmer or more brackish waters (e.g., *Striatolamia, Carcharias*) or deeper, colder waters (e.g., *Pristiophorus*). Taxa like *Raja* and *Squalus* display no shift, tracking local conditions in Seymour Island. The lack of difference in δ^18^O_p_ values between pelagic and benthic sharks in the Late Eocene could suggest a poorly stratified water column, inconsistent with a fully opened Drake Passage. Our findings demonstrate that shark tooth bioapatite tracks the preferred habitat conditions for individual taxa rather than recording environmental conditions where they are found. A lack of secular variation in δ^18^O_p_ values says more about species ecology than the absence of regional or global environmental changes.

## 1 Introduction

Sharks and rays (Chondrichthyes: Elasmobranchii) have thrived in marine environments during periods of climate change throughout geological time. Their success is documented in their fossil record mostly as isolated teeth, which accumulate in ocean sediments and indirectly record their evolutionary history and the environ-mental conditions in which they lived (Cappetta, 2012; Condamine et al., 2019; Vennemann et al., 2001). Elasmobranchs have survived four major mass extinction events (Benton, 2014; Cappetta, 2012), with the wax and wane of clades shaped by some combination of clade competition (Condamine et al., 2019), niche adaptation (Bazzi et al., 2021), or global temperature changes (Condamine et al., 2019) based on correlation with benthic foraminifera isotope records (Zachos et al., 2008). While these studies focus on patterns of diversity and tooth morphology, exploring the environmental conditions that elasmobranchs experienced during these periods of global climate change could shed light on their ecological plasticity over geological time.

The Eocene Epoch (56–33.9 Ma) was characterized by major climatic shifts within the Cenozoic Era (Zachos et al., 2001, 2008) and elasmobranchs were spatially abundant spanning the Arctic to Antarctic (Cappetta, 2012; Kim et al., 2022). Geochemical proxies and climate simulations suggest that these temperature shifts were caused by some combination of a decrease in *p*CO_2_ levels (DeConto & Pollard, 2003; Hönisch et al., 2023) and the opening of oceanic gateways (Kennett, 1977; Kennett & Exon, 2004) approaching the Eocene-Oligocene transition (EOT), setting the stage for a shift from a Greenhouse to an Icehouse world (Zachos et al., 1996, 2001). The widening of the Tasman Gateway and the Drake Passage could have enabled the formation of cold currents around Antarctica (i.e., Antarctic Circumpolar Currents, ACC) and eventually facilitated the rapid establishment of ice caps during the EOT (Egan et al., 2013; Kennett, 1977; Kennett & Exon, 2004; Scher & Martin, 2004, 2006; Scher et al., 2011; Stickley et al., 2004). While the Tasman Gateway opened to deep circulation between 35.5 and 33.3 Ma (Stickley et al., 2004), the timing of the Drake Passage deepening raises uncertainties about its impact on climate and strength of an ACC-like current. Geological and geochemical evidence suggests that the sector began opening at shallow depths ~41 Ma, continuing to widen throughout the Eocene (Eagles et al., 2006; Kim et al., 2020; Lagabrielle et al., 2009; Scher & Martin, 2006). However, many authors argue the opening to deep eastward circulation in the Drake Passage likely occurred ~32 Ma (Eagles et al., 2006; Hodel et al., 2021; Lagabrielle et al., 2009; Latimer & Filippelli, 2002; Lawver & Gahagan, 2003)—the degree to which the opening of the Drake Passage contributed to global cooling during the Eocene might be better evaluated by information on the thermal structure of the water column. Several simulations with varying complexity, resolution, and boundary conditions agree that deepening the Drake Passage reduces poleward heat transport making sea surface waters colder and saltier. However, the magnitude of this shift in density structures (both temperature and salinity changes) due to tectonic deepening varies, with some studies suggesting minimal influence (Goldner et al., 2014; Zhang et al., 2010) and others indicating a moderate impact (Nong et al., 2000; Sijp & England, 2004). Additionally, the deepening of gateways could impact regional and global climate without promoting the formation of an ACC current that thermally isolates the continent like modern-day scenarios (Sauermilch et al., 2021). To better predict the impact on climate related to the opening of the Drake Passage throughout the Eocene, more empirical observations accounting for patterns of surface and deeper waters within this sector are necessary, given that current records are sparse.

Because Eocene global climate change impacted flora and fauna, including elasmobranchs in high-latitude areas (Cione et al., 2007; Condamine et al., 2019; Egan et al., 2013; Gelfo et al., 2019; Ivany et al., 2000; Kim et al., 2020; Krug et al., 2010; Millar, 1993; Mörs et al., 2020), their fossil record is likely to offer insight into the timing of and mechanisms for cooling. Analyzing the geochemistry of elasmobranch teeth can provide valuable insights into their habitat use to assess their resilience to climate change. In addition, because some elasmobranchs are highly mobile and could conceivably have traveled well offshore into deeper water (Compagno, 2002), the difference between pelagic and benthic elasmobranchs can also provide insight into the structure of the water column and hence into the configuration of the Drake Passage during the Eocene.

**Figure 1:**
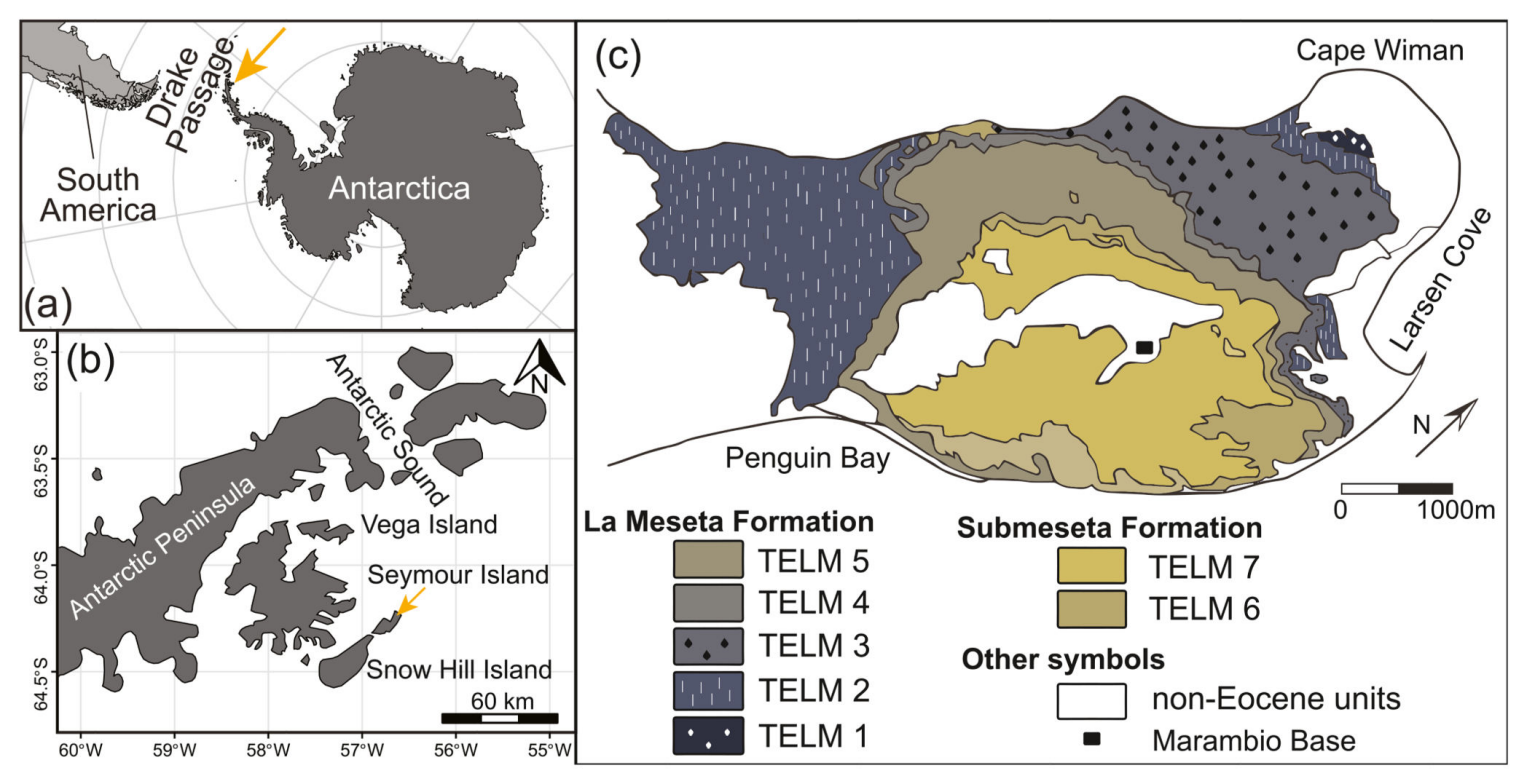
(a) Location of Seymour Island (yellow arrow) in relation to the Drake Passage, South America, and (b) other islands around the Antarctic Peninsula. (c) Geographic distribution of TELMs on Seymour Island.

The Eocene fossil record in Antarctica offers context to elucidate habitat use and environmental conditions experienced by elasmobranchs during the time leading up to glaciation. In this study, we seek to explore the interplay between elasmobranch ecology and environmental shifts from fossil shark teeth collected from the Middle to Late Eocene, in shallow marine deposits of the La Meseta Formation and estuarine-coastal deposits of the Submeseta Formation (Amenábar et al., 2020; Marenssi et al., 1998; Montes et al., 2013; Porębski, 2000; Sadler, 1988). These nearshore Eocene deposits are located in Seymour Island (James Ross Basin, Weddell Sea, West Antarctica), an area ~600 km south of the Drake Passage (Figure 1a) that experienced warmer sea surface temperatures (10–17°C) than today (~0°C) during the Eocene (Douglas et al., 2014; Huck et al., 2017; Ivany et al., 2008; Judd et al., 2019; Kim et al., 2020; Langton et al., 2016; Zachos et al., 2008). The stratigraphical units of these deposits capture a variably diverse chondrichthyan fossil record, comprised of isolated teeth that are the focus of extensive taxonomic identification during the last 40 years (Engelbrecht et al., 2016a, 2016b, 2017a, 2017b; Kriwet, 2005; Kriwet et al., 2016; Long, 1992b; Marramá et al., 2018; Porębski, 2000; Welton & Zinsmeister, 1980). The taxonomy of fossil sharks and rays from Seymour Island suggests variations in their habitat preferences, but tooth geochemistry could provide further insights into habitat use, as well as ocean chemistry and temperature (Kim et al., 2020; Vennemann et al., 2001; Zacke et al., 2009).

The oxygen isotope composition of elasmobranch teeth (δ^18^O) preserves the environmental conditions in agreement with the lifestyles of individuals. The δ^18^O values of elasmobranch tooth enameloid and dentin bioapatite depend on water composition (δ^18^O_w_), which is controlled by global ice volume modified by local to regional salinity, and body temperature, which is assumed to approximate water temperature in ectothermic sharks and rays. Teeth incorporate oxygen starting from the initial stages of tooth mineralization, and oxygen isotope fractionation between body water and bioapatite is temperature-dependent in both phosphate and carbonate substrates (Kemp, 1985; Kohn & Cerling, 2003; Lécuyer et al., 2013; Longinelli & Nuti, 1973a, 1973b; Shemesh et al., 1983; Vennemann et al., 2002). In elasmobranch teeth, the phosphate component of the bioapatite is preferred for measuring δ^18^O values (δ^18^O_p_) due to its resistance to diagenetic alteration unlike other oxygen substrates, such as structural carbonate (Enax et al., 2012; Zazzo et al., 2004). While teeth develop, the bioapatite records snapshot seawater temperature and salinity conditions along the direction of mineralization (i.e., from the apex to the basal portion of the crown in enameloid, outer to inner portion in the dentin) (Jambura et al., 2018; Vennemann et al., 2001; Žigaite & Whitehouse, 2014). Teeth move forward in the jaw in a conveyor belt motion and become fully mineralized once they reach the functional position (Jambura et al., 2018). The time elapsed between tooth formation and shedding is species-specific and could be relatively long. For example, tooth replacement rates per row in *Negaprion brevirostris, Triakis semifasciata*, and *Carcharodon carcharias* are 8, 55, and 227 days, respectively (Botella et al., 2009; Bruner, 1998; Kim et al., 2012). As teeth mineralize, shark individuals experience variable environmental conditions across time and space. Chondrichthyans demonstrate diverse life history strategies, with some species living in the water column (pelagic) and others on the ocean floor near the coast out to the continental slope (benthic). Some taxa inhabit more restricted areas with less movement, while others undergo extensive seasonal migration across latitudinal or depth gradients (Compagno, 2002; Kneebone et al., 2014; Sulikowski et al., 2010). Therefore, δ^18^O_p_ values of elasmobranchs likely record preferred environmental conditions across broader or narrower areas dependent upon the combination of their lifestyles and tooth replacement rates.

Here, we characterize habitat use and environmental conditions captured in elasmobranch tooth δ^18^O_p_ values and compare the oxygen isotope composition of elasmobranchs with those of co-occurring bivalves as well as predictions from isotope-enabled climate model simulations. First, we assess whether the composition of elasmobranch teeth from the Eocene deposits of Seymour Island reflects a local climatic signal. If so, we expect an increase in mean and median δ^18^O_p_ values from older to younger deposits similar to δ^18^O_c_ values from co-occurring bivalves (*Cucullaea* and *Retrotapes*) (Ivany et al., 2008). These bivalve taxa, as infaunal individuals inhabiting the benthos of the Seymour Island marine shelf, provide a point of comparison with elasmobranchs, which are vagile and hence capable of moving among environments. Second, we evaluate whether elasmobranch teeth capture seasonal or spatial variability in their δ^18^O values, a hypothesis that was previously proposed for *Striatolamia macrota* (Kim et al., 2020). We use water temperature and δ^18^O_w_ values from model outputs to predict δ^18^O_p_ values (denoted as δ^18^O_p_*) using the isotope-enabled Community Earth System Model version 1.2 for the Early Eocene (iCESM; Zhu et al., 2020). If elasmobranch taxa remain in the region and in the same habitats year-long, the range of variation in their teeth will match seasonal δ^18^O_p_* values for Seymour Island (i.e., local environmental signal). Alternatively, a wider range in δ^18^O_p_ values than those predicted by the climatic model would indicate that elasmobranchs range widely across the suite of habitats around the Peninsula, while a narrower range in tooth bioapatite suggests instead that they track their preferred temperatures across the region as seasonal and secular temperature change unfolds. Lastly, we explore whether elasmobranchs experienced shifts in habitat use in response to changes in thermal stratification in the ocean due to tectonic changes in the Drake Passage throughout the Middle to the Late Eocene. If the deepening of the Drake Passage is involved in Eocene cooling, regional conditions would likely reflect the broader shift toward enhanced thermal stratification associated with the developing ACC (Hodel et al., 2021). We hypothesize that at least some teeth from benthic elasmobranchs would record colder water indicated by higher δ^18^O_p_ values compared to pelagic relatives toward the end of the Eocene. If so, benthic elasmobranchs would also have higher δ^18^O_p_ values than predicted δ^18^O_p_* and the δ^18^O of co-occurring bivalves and model simulations for Seymour Island (Ivany et al., 2008; Zhu et al., 2020). This shift could result from the deepening of the Drake Passage and colder conditions at depth relative to surface water (Hodel et al., 2021), which would be offshore areas inhabited by benthic sharks.

## 2 Geological Setting

The La Meseta (LMF) and Submeseta formations (SMF) crop out on Seymour Island, which is located east of the Antarctica Peninsula within the James Ross Basin at 64°17′S, 56°45′W (Figure 1) (Dutton et al., 2002; Elliot, 1988). The sedimentary succession forms the Seymour Island Group together with the Middle-Upper Paleocene Cross Valley Formation and overlays the Upper Cretaceous-Lower Paleocene Marambio Group (Gaździcki et al., 1992; Ivany et al., 2008; Marenssi et al., 1998; Porębski, 2000). The LMF and SMF include shell beds, siltstones, and sandstones that are deposited in shallow coastal and estuarine environments. The depositional motif repeats in seven lithofacies known as Tertiary Eocene La Meseta units (TELMs, Figure 1c), which are bounded by angular unconformities and are categorized by biostratigraphy (Marenssi et al., 1998; Porębski, 2000; Sadler, 1988). The succession is further divided into six allomembers based on the relationship between lithology and facies: Valle de Las Focas (TELM 1), Acantilados I (TELM 2), Acantilados II/Campamento (TELM 3), Cucullaea I and II (TELMs 4 and 5). The Submeseta Allomember was formerly used to describe the top of the sedimentary succession included in TELM 6 and 7, but some now consider it to be a new formation (i.e., Sub-meseta Formation) (Marenssi et al., 1998; Montes et al., 2013). While the stratigraphic position of the Eocene TELMs is well established, the absolute age model of the units has been frequently revised and is still uncertain. Previous ^87^Sr/^86^Sr measurements on carbonate of co-occurring bivalves tentatively assigned the deposition of TELM 2 to 3 to 55–51 Ma (i.e., Ypresian age, Early Eocene) (Ivany et al., 2006, 2008). However, uncertainties in the global marine strontium-isotope seawater curve for the Early to Middle Eocene (McArthur et al., 2001), combined with the incorporation of external, local strontium sources in co-occurring bivalves (e.g., freshwater mixing), confound the geochronological positioning of TELM units at the bottom of the sedimentary succession when using this approach (Douglas et al., 2014). Despite this uncertainty, robust biostratigraphic and isotope analyses suggest deposition between the Middle and Late Eocene for the lithofacies: (a) the biostratigraphy of TELMs units (Amenábar et al., 2020; Douglas et al., 2014) match the dinoflagellate cyst zonation SPDZ 10 to 13 applied for the Southern Ocean across the Middle and Late Eocene, calibrated with magnetostratigraphy (Chron C20n to C13n) (Bijl et al., 2013); (b) ^87^Sr/^86^Sr chemostratigraphy analyses on co-occurring bivalves from SMF match the global marine strontium-isotope seawater curve (Douglas et al., 2014; Ivany et al., 2006, 2008); (c) neodymium isotope measurements (^143^Nd/^144^Nd, ε_Nd_) from *S. macrota* teeth collected from LMF deposits reflect shifts toward more radiogenic values observed in sediments from deep-sea sites IODP 689 and 1090 during the Middle Eocene (Scher & Martin, 2006). Given this evidence, the uncertainty in the absolute age models does not significantly impact the paleoecological and environmental interpretations presented in this study.

The shark tooth specimens preserved in the Eocene deposits of Seymour Island are common in TELM 2 to 6, with TELM 4 and 5 having the highest abundance and richness of taxa while isolated teeth are rare in TELM 1 and 7 (Engelbrecht et al., 2016a, 2016b, 2017a, 2017b, 2019; Kriwet, 2005; Kriwet et al., 2016; Long, 1992b; Marramá et al., 2018). The age model for these lithofacies is summarized in Kim et al. (2020), which combines the updated biostratigraphy based on dinoflagellate cyst content (Amenábar et al., 2020) and ^87^Sr/^86^Sr chemostratigraphy analyses after Douglas et al. (2014) and Ivany et al. (2006, 2008). The lower unit of the LMF (TELM 1) is marked by the alternate occurrence of the endemic, “Transantarctic” *Enneadocysta dictyostila* and *Deflandrea antarctica*, which is calibrated to ~46– ~45 Ma (Amenábar et al., 2020; Douglas et al., 2014). The endemic fauna found in TELM 2 and 3 (*E. dictyostila, Arachnodinium antarcticum*, and *Hystricosphaerodoim truswelliae*) suggests deposition between ~45 Ma and ~38 Ma (Amenábar et al., 2020; Douglas et al., 2014). In TELM 4, the palynological content is similar to that of the lower TELM 3 unit but the unique occurrence of *Deflandrea granulata* and the presence of *Glaphyrocysta semitecta* and *Deflandrea cygniformis* at the bottom of overlying TELM 5 indicates deposition between ~41 and 39.10 Ma (Amenábar et al., 2020; Douglas et al., 2014). The upper section of the LMF (TELM 5) was deposited between ~41 and ~37 Ma as suggested by the occurrence of *E. dictyostila, Alterbidinium distinctum, Brigantedinium* spp., *Lejeunecysta* spp., and *Selenopemphix nephroides* (Amenábar et al., 2020; Douglas et al., 2014). The palynological content in TELM units from LMF suggests coeval deposition with the Eocene deposits from the Río Turbo, Río Baguales, and Loreto formations that outcrop in Chile (Sierra Dorotea, Río Baguales, and Río de Las Minas; Magallanes Basin); all formations preserve an elasmobranch fossil fauna similar in genus richness to that found in LFM (Amenábar et al., 2020; Estebenet et al., 2017; Kriwet et al., 2016; Otero & Soto-Acuña, 2015b; Otero et al., 2012, 2013). The Submeseta Formation includes TELM 6 and 7, where ^87^Sr/^86^Sr chemostratigraphy estimated an age of ~41 Ma or younger for the lower and immediately before the Eocene-Oligocene Transition (EOT) for the upper unit, respectively (Douglas et al., 2014; Ivany et al., 2006). To summarize, the shark assemblage found in LMF and SMF was likely formed between the Middle Lutetian (~46 Ma, Middle Eocene) and the Late Priabonian (~34 Ma, Late Eocene).

## 3 Materials and Methods

### 3.1 Material

The elasmobranch fossil material was collected as isolated teeth specimens from Eocene TELMs 2 to 7 during the 2011–2013 summer campaigns led by the collaboration of the Instituto Antàrctico Argentino (DNA-IAA) and the Swedish Polar Research Secretary (SPFS) on Seymour Island. We analyzed 201 tooth specimens from the Paleozoological Collection of the Swedish Museum of Natural History (NRM-PZ; Stockholm, Sweden). All taxa were assigned to pelagic and benthic habitats, respectively, based on modern analogs (*n* species = 10) determined by tooth morphology and taxonomy. Fossil taxa classified into the genera *Carcharias, Pristiophorus*, and *Squalus* are more closely related to their modern representatives compared to other fossil-modern analogies (Table 1) (Cunningham, 2000; Engelbrecht et al., 2016a, 2017a, 2017b, 2019; Kriwet et al., 2016; Long, 1992a; Marramá et al., 2018; Parmley et al., 2003). We summarize in Table 1 the taxa featured in this study with specimens per TELM along with the expected habitat, temperature, and depth ranges based on modern analogs. Temperature variations in modern analogs reflect a combination of spatial gradients (such as latitudinal and depth transects) and seasonal environmental variations in a single locality. This occurs because sharks and rays move to preferred environmental conditions (e.g., Compagno, 1984; Kneebone et al., 2014; Sulikowski et al., 2010). Environmental preferences were inferred based on AquaMaps, FishBase, and published literature on modern analogs (Compagno, 2002; Froese & Pauly, 2024; Kaschner et al., 2015; Kneebone et al., 2014; Sulikowski et al., 2010; Weigmann, 2016). In addition to the material analyzed here (Table 1), we included *S. macrota* tooth specimens collected from TELMs 2 to 5 (*n* = 42) analyzed in Kim et al. (2020) and Zeichner et al. (2015), a taxon displaying morphological similarities to the modern sand tiger shark *Carcharias taurus* (Cunningham, 2000). We note that expected habitat preferences are based on taxonomy comparisons and may not necessarily reflect the actual temperatures or depths preferred by fossil taxa in Seymour Island across the Eocene.

### 3.2 Analytical Techniques

#### 3.2.1 Stable Oxygen Isotope Analysis

We used two different protocols to precipitate silver phosphate (Ag_3_PO_4_) from elasmobranch tooth bioapatite based on specimen size. Larger specimens (i.e., crown height >2 cm) where enameloid could be drilled were prepared following Mine et al. (2017) whereas smaller specimens requiring crushing were prepared following Larocca Conte, Lopes, et al. (2024); an account of the decision-making process and description of the silver phosphate precipitation protocols are detailed in Supporting Information (SI1) in Supporting Information S1. Briefly, biological bioapatite was dissolved in hydrofluoric acid, phosphate ions isolated in solution, and silver phosphate precipitated with a silver amine solution. Silver phosphate crystals were rinsed five times with deionized water and dried overnight at 50°C. Triplicate analyses of ~0.2 mg Ag_3_PO_4_ per specimen were packed into silver capsules and run in a Thermal Conversion Elemental Analyzer (TC/EA)-ConFlo IV-Delta V Plus continuous flow isotope ratio mass spectrometer system (Thermo Scientific, Bremen, Germany) at the Stable Isotope Ecosystem Laboratory of (SIELO) University of California, Merced (California, USA). Silver phosphate reduction to CO gas was achieved by heating the TC/EA graphite column at 1,450°C. Raw measurements were corrected for drift and linearity effects using the silver phosphate reference materials USGS 80 and USGS 81 (USGS; >99% purity). A 2-point calibration was applied to calibrate corrected δ^18^O_p_ measurements to the Vienna Standard Mean Ocean Water scale (V-SMOW) using the same Ag_3_PO_4_ reference materials (USGS 80 δ^18^O_p_ = 13.1%o, USGS 81 δ^18^O_p_ = 35.4%o). Analytical precision within runs (*n* = 19) ranged from 0.1 to 0.4%o for USGS 80 and from 0.2 to 0.4%o for USGS 81, while the analytical uncertainty across runs is 0.3%o for both reference materials (USGS 80 *n* = 237, USGS 81 *n* = 228). Shark and ray tooth δ^18^O_p_ values are reported as mean ± 1σ.

#### 3.2.2 Fourier-Transform Infrared Spectroscopy (FTIR)

In addition to isotopic composition, we tested the extent of diagenetic alteration in a selected group of shark tooth specimens (*n* = 14) via Fourier-Transform Infrared analysis (FTIR). Although the selected specimens do not cover all taxa studied, we chose specimens with varying degrees of preservation (e.g., whole teeth vs. specimens with only crown height) and materials (enameloid only vs. enameloid-dentin mixtures). This approach was intended to assess the degree of post-burial alteration and provide context for biological-environmental signals recorded in δ^18^O_p_ values of elasmobranchs. We obtained spectra in the 400–4,000 cm^−1^ range using a Bruker Vertex 70 Far-Infrared in ATR mode, located at the Nuclear Magnetic Resonance Facility at the University of California, Merced (California, USA). Each sample’s spectra underwent smoothing by averaging 32 scans with a 4 cm^−1^ resolution each. We applied posterior baseline correction to align infrared peaks on points where absorbance intensity is expected to be 0. We fitted and subtracted spline curves to these points to obtain a flat baseline (Trayler et al., 2023).

## 4 Data Analysis

We built a framework with model simulations and empirical measurements that comprises independent sets of oxygen isotope values aiming to explain ecological and environmental signals measured from shark tooth δ^18^O_p_ values. We analyzed the probability density distribution of bulk elasmobranchs’ δ^18^O_p_ measurements across TELMs to evaluate shifts in their central tendency across time. Statistical significance between TELMs was evaluated with a Kruskal-Wallis test and post-hoc Dunn test analysis for pairwise comparisons between different stratigraphic units.

We explored possible seasonal and spatial signals embedded in elasmobranchs’ δ^18^O_p_ values by comparing measurements with predictions from a global climate model. We used the Early Eocene isotope-enabled iCESM 1.2 simulations outputs of temperature and δ^18^O_w_ composition (Brady et al., 2019; Zhu et al., 2019, 2020) to predict δ^18^O_p_* values. The model is calibrated with paleogeographic boundary conditions for the Early Eocene (Herold et al., 2014), which includes the Drake Passage and the Tasman Gateway open to shallow, epicontinental circulation in the Southern Ocean (i.e., bathymetry set to <100 and 30 m b.s.l., respectively). This early Eocene paleogeography is used for all simulations, so while it reflects the general conditions of the time, it may not perfectly align with the specific paleogeographic features of the Middle to Late Eocene study interval. However, the model includes simulations with different climate states, allowing us to test environmental settings across the Eocene Epoch. We used offline outputs of temperature and δ^18^O_w_ values using simulations equilibrated at 3× and 6× pre-industrial CO_2_ levels (i.e., 284.7 ppmv) within a water column of 25 m. We averaged the last 100-year simulations per climatic state. To define which climatic state would best represent seasonal environmental trends in Seymour Island, we compared monthly averaged model outputs with serial δ^18^O_c_ values from the co-occurring bivalves *Cucullaea* and *Retrotapes* (Buick & Ivany, 2004; Ivany et al., 2008; Judd et al., 2019). Serial δ^18^O_c_ measurements within single bivalve specimens (*n* = 8) provide high-resolution seasonal trends in Seymour Island; these previously published bivalve data were only available for TELM 5. Model δ^18^O_c_ estimates (δ^18^O_c_*) were computed using the Grossman and Ku (1986) paleothermometer equation for aragonitic shells, solving Equation 1 for δ^18^O_c_:

(1) $$\text{T}({}^{\circ}\text{C})=20.6-4.34*(\delta^{18}{\text{O}}_{\text{c}V-\text{PDB}}-\delta^{18}{\text{O}}_{\text{W}\;\text{V-SMOW}})$$

where T is the temperature, δ^18^O_c_ (in V-PDB), and δ^18^O_w_ are carbonate (i.e., aragonite) and water oxygen isotope compositions.

Following, we explored the habitat use of elasmobranch taxa and estimated their temperature preferences to inform whether δ^18^O_p_ values from each taxon would reflect local or regional environmental signals. We compared density distributions for δ^18^O_p_ values from pelagic and benthic taxa with predicted seasonal and regional δ^18^O_p_* values from the iCESM simulation and co-occurring bivalves’ bulk δ^18^O_c_ values (Ivany et al., 2008; Zhu et al., 2020). We used the recalibrated paleothermometer equation of Kolodny et al. (1983) in Lécuyer et al. (2013) to compute forward δ^18^O_p_* predictions and solved for δ^18^O_p_:

(2) $$\text{T}({}^{\circ}\text{C})=117.4(\pm 9.5)-4.50(\pm 0.43)*(\delta^{18}{\text{O}}_{\text{p}\;\text{V-SMOW}}-\delta^{18}{\text{O}}_{\text{W}\;\text{V-SMOW}})$$

where T is the estimated temperature in Celsius degrees given the fractionation between phosphate (δ^18^O_p_) and modeled water oxygen isotope composition (δ^18^O_w_; Zhu et al., 2020). We preferred to use this equation over others found in literature (Kolodny et al., 1983; Longinelli & Nuti, 1973b; Pucéat et al., 2010) because: (a) it performs comparably to other paleothermometer equations (Figure SI4.1 in Supporting Information S1), except for the Pucéat et al. (2010) equation, which generates estimates higher by a few per mil compared to other oxygen isotope fractionation equations (Figure SI4.1 in Supporting Information S1). These differences likely arose from experimental errors, as the Pucéat et al. equation was derived from controlled experiments on fish in aquaria, where the isotopic composition of the water was not constant, leading to error in the determination of the oxygen-isotope fractionation equation (Lécuyer et al., 2013; Pucéat et al., 2010); (b) it provides temperature values from fish tooth specimens consistent with estimates of co-existing bivalves or brachiopod carbonate shells (Lécuyer et al., 2013). Estimated δ^18^O_p_* values from co-occurring bivalves were obtained after applying the following transfer Functions 3 and 4 (Ivany et al., 2008; Kim et al., 2015; Longinelli & Nuti, 1973b; Zhu et al., 2020):

(3) $${\text{δ}}^{18}{\text{O}}_{\text{c}\;\text{V-SMOW}}\text{30}.92+1.03092*{\text{δ}}^{18}{\text{O}}_{\text{c}\;\text{V}-\text{PDB}}$$

(4) $${\text{δ}}^{18}{\text{O}}_{\text{P}\;\text{V-SMOW}}\text{=}\frac{{\text{δ}}^{18}{\text{O}}_{\text{c}\;\text{V-SMOW}}-8.\;67\;(\pm 1.\;24)}{1.02\;(\pm 0.06)}$$

These equations involve first the conversion of δ^18^O_c_ values from V-PDB to V-SMOW scale (Equation 3; Kim et al., 2015) and subsequent transformation into δ^18^O_p_* values using Equation 4 (*R*^2^ = 0.93) after the oxygen isotope measurements for both carbonate and phosphate components in invertebrates (*n* = 27; Figure SI5.2 in Supporting Information S1; Longinelli and Nuti, 1973b). These estimates from bivalves’ bulk δ^18^O_c_ measurements were compared with δ^18^O_p_ values of sharks and rays as a supplementary marker for local conditions in Seymour Island along with predictions from the iCESM model, which are in good agreement with seasonal simulated values for Seymour Island (see Section 6.2, Tables SI4.2 and SI5.2 in Supporting Information S1).

We determined temperature represented by pelagic and benthic taxa using a Bayesian approach similar to that of Griffith et al. (2023; full description is found in Supporting Information SI3 in Supporting Information S1). This framework attempts to estimate probable environmental temperature using model outputs as prior information (i.e., simulated temperature and δ^18^O_w_, discussed above), assuming that sharks and rays do not necessarily inhabit unique environments (Table 1). We integrated Equation 2 in the Bayesian model to estimate temperature from elasmobranchs’ δ^18^O_p_ values.

Finally, we compared the central tendency observed from δ^18^O_p_ values in pelagic and benthic elasmobranchs to explore shifts in habitat use between groups. This shift could result from deepening of the Drake Passage and stronger thermal stratifications between surface and deeper waters, which are habitats that pelagic and benthic elasmobranchs would inhabit, respectively. We used the Kruskal-Wallis test to quantify statistical differences between groups per TELM unit.

All data sets were analyzed in R Studio (R Development Core Team, 2024). Model outputs are extracted using XArray, MatplotLib, and Cartopy libraries in Python before their analysis (Hoyer & Joseph, 2017; Hunter, 2007; Met Office, 2015).

## 5 Results

### 5.1 Elasmobranch Bioapatite δ^18^O_p_ Values

**Figure 2:**
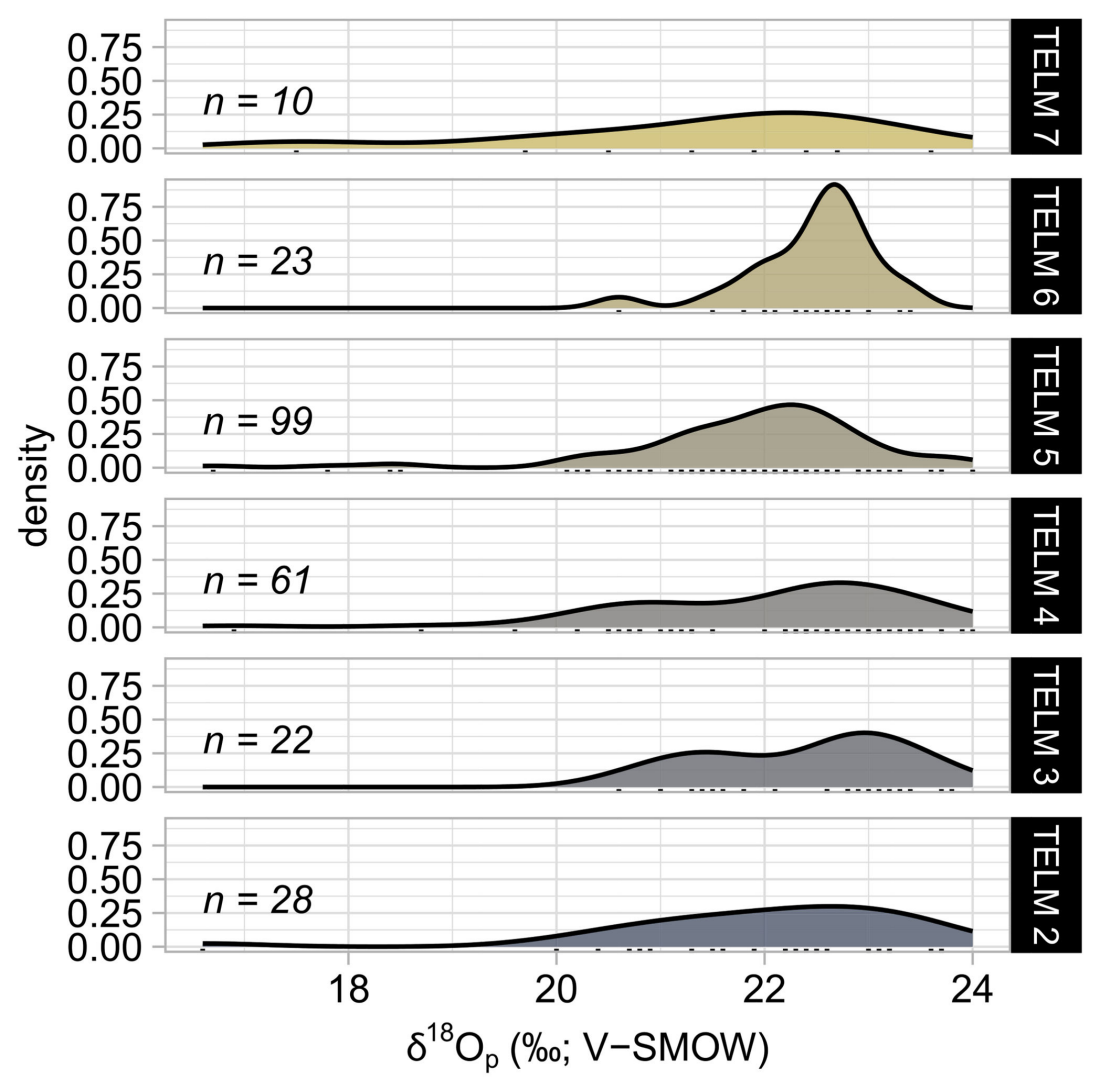
Elasmobranchs’ δ^18^O_p_ values suggest environmental stasis across TELMs. The figure shows the density distribution of δ^18^O_p_ values measured from elasmobranch tooth specimens collected from LMF (TELM 2 to 5) and SMF (TELM 6 and 7). Colors indicate sharks’ and rays’ δ^18^O_p_ distributions in different TELMs.

Phosphate oxygen isotope values measured from elasmobranch teeth have a mean value of 22.0 ± 1.3%o (*n* = 243). Bulk δ^18^O_p_ distributions exhibit skewed shapes and a similar broad variability across TELMs (Figure 2, Table 2). Mean δ^18^O_p_ varies from 22.0 ± 1.5%o in TELM 2 (*n* = 28; Middle Eocene) to 21.4 ± 1.8%o in TELM 7 (*n* = 10; Late Eocene) (Table 2). Generally, the median δ^18^O_p_ value is a few per mil higher than the mean values across TELMs, but the distributions are similar across units (Table 2). A Kruskal-Wallis test indicates no statistically significant differences in the distributions between TELMs (*H* = 6.81, df = 5, *p* = 0.07). Post-hoc Dunn Test suggests that δ^18^O_p_ values across TELMs remain relatively stable (*p* < 0.05), with TELM 5 and 6 being the only units showing statistical dissimilarity (*p* > 0.05; Table 3). Although the mean δ^18^O_p_ value notably increases from 21.9 ± 1.2%o to 22.5 ± 0.6%o across these units, TELM 5 and 6 do not exhibit statistical differences with any other TELM (Table 3), and this statistical result may be due to sample size. In summary, bulk δ^18^O_p_ values of shark and ray teeth cannot establish significant changes throughout TELMs.

### 5.2 iCESM Model Validation for Seasonal Trends in Seymour Island: 3× Versus 6× Pre-Industrial CO_2_ Level Simulations

**Figure 3:**
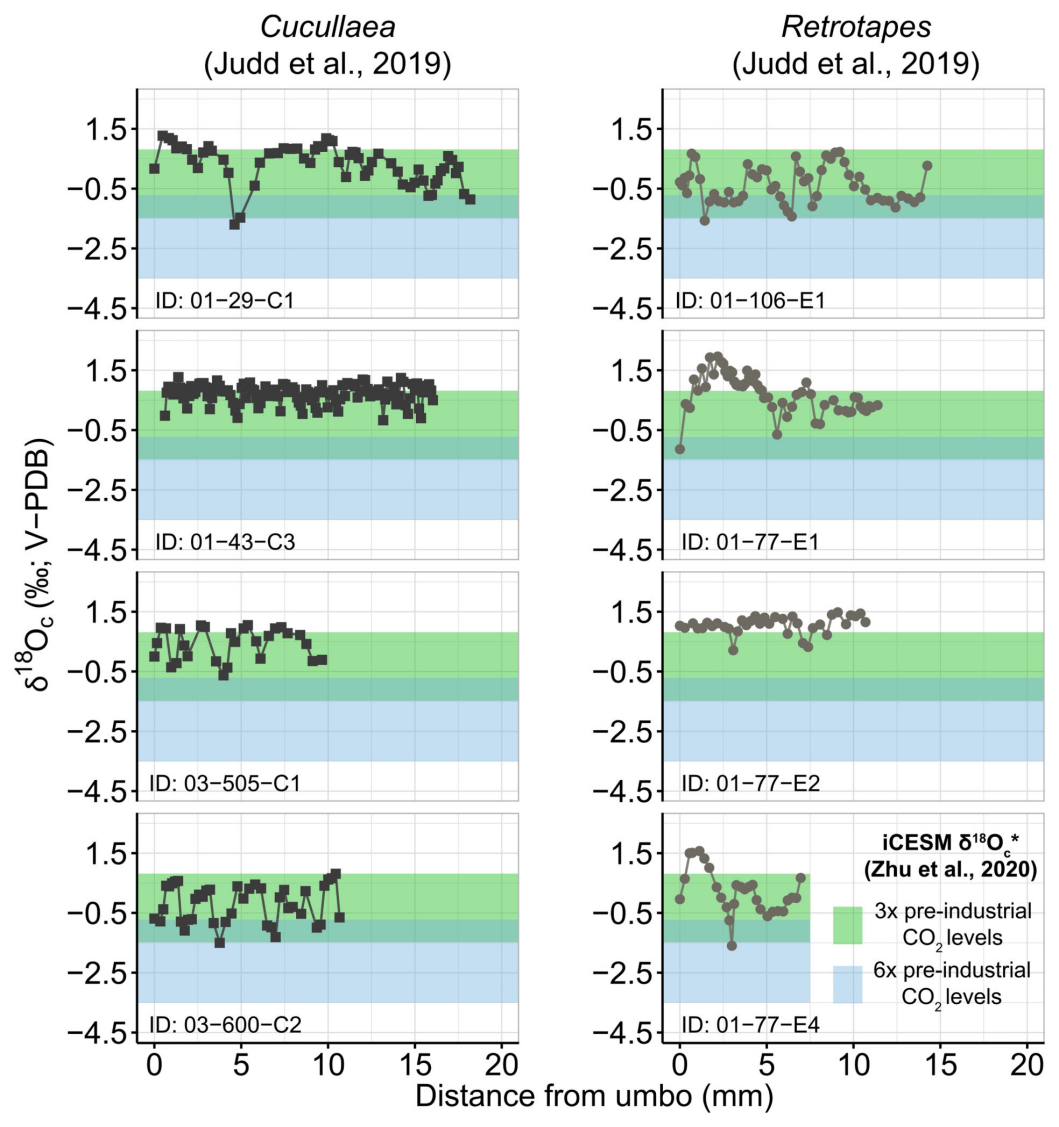
δ^18^O_c_ values from individual serial sampled bivalve specimens (Judd et al., 2019) compared to iCESM simulated values for Seymour Island integrated from surface to 25 m (Zhu et al., 2020). The 3× pre-industrial CO_2_ case predicts seasonal environmental variation for Seymour Island in excellent agreement with empirical. The plot shows δ^18^O_c_ values of *Cucullaea* (left panel, black squares) and *Retrotapes* (right panel, gray circles) individuals measured along the shell, from the umbo to the outer edge of specimens. The range of simulated monthly averaged δ^18^O_c_* values are shown with the green and light blue boxes for the 3× and 6× CO_2_ cases, respectively.

Seasonal variation in Early Eocene environmental conditions at Seymour Island is represented by monthly averaged δ^18^O_c_* values estimated from the iCESM simulations within a 25 m sea-surface column (Zhu et al., 2020) (Table SI4.1 in Supporting Information S1). Model δ^18^O_c_* ranges vary based on *p*CO_2_ conditions: for 3× pre-industrial levels, δ^18^O_c_*are - 1.5 to 0.8%o and for 6× pre-industrial levels, δ^18^O_c_* are −3.5 to −0.7%o (Figure 3), corresponding to the austral summer and winter temperature peaks in March and September–October (Table SI5.1 in Supporting Information S1). When simulated values are compared to serial δ^18^O_c_ values of the bivalves *Cucullaea* and *Retrotapes* (Judd et al., 2019), δ^18^O_c_* values for the 6× CO_2_ case are too low (Figure 3). Although δ^18^O_c_ measurements in *Cucullaea* and *Retrotapes* specimens sometimes exceed simulated values by up to 1.1%o (*Retrotapes* specimen 01-77-E1; Figure 3), these instances of proxy-model mismatch are few. Overall, simulated δ^18^O_c_* values for the 3× CO_2_ case have a better correspondence with seasonal environmental conditions for Seymour Island during the Eocene, aligning well with empirical data measured from mollusks. Furthermore, simulated environmental conditions under such CO_2_ boundary levels well agree with current *p*CO_2_ estimates from benthic foraminifera and biomarkers for the Middle-Late Eocene (i.e., ~800 p.p.m.; Anagnostou et al., 2016; Pearson et al., 2009; Zachos et al., 2008).

### 5.3 Comparisons With Seasonal Trends From iCESM Model at Seymour Island

**Figure 4:**
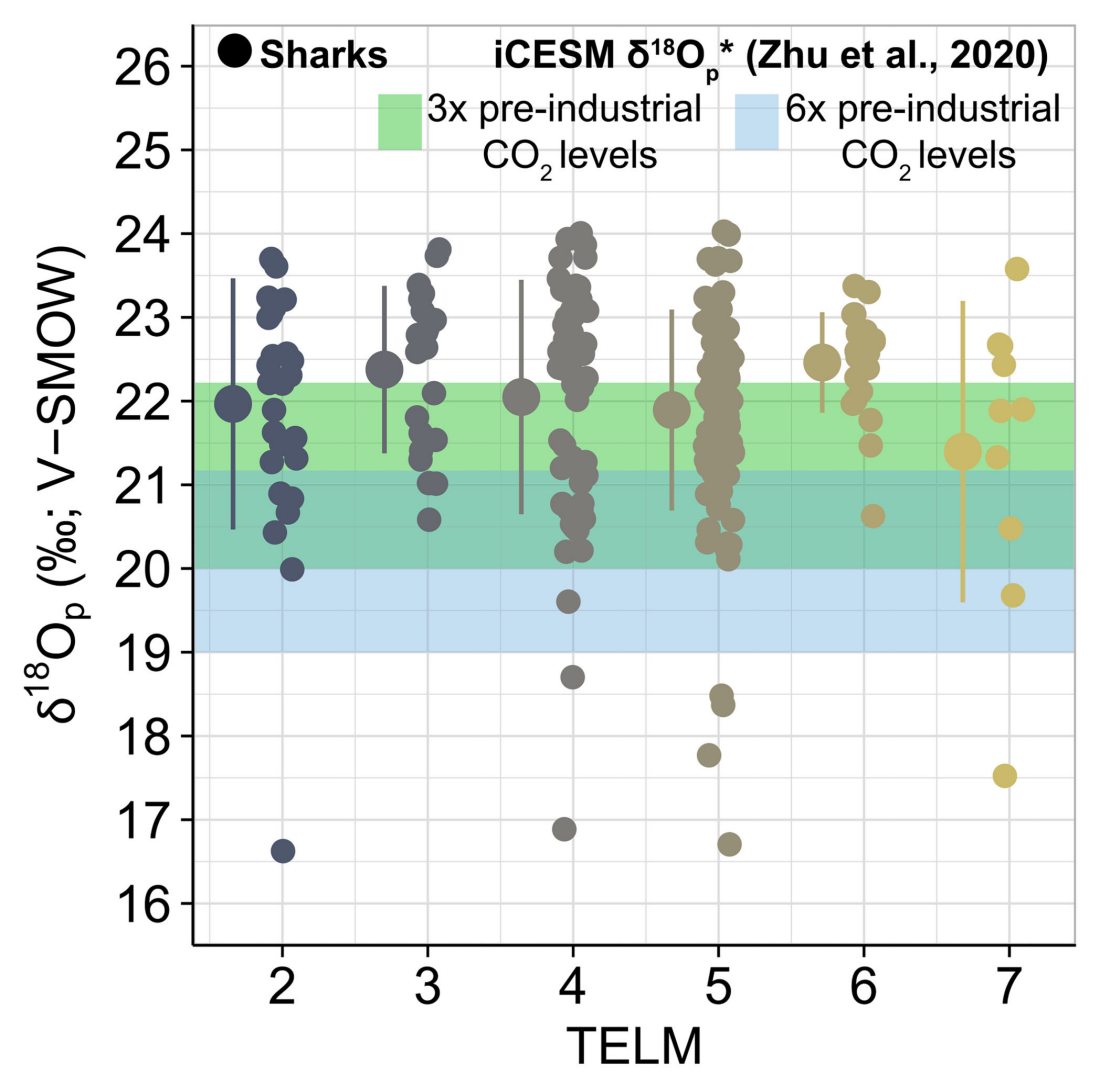
Bulk elasmobranch bioapatite δ^18^O_p_ values exhibit a large variation across TELMs, which spans predicted Eocene seasonal values for Seymour Island. Colors for sharks δ^18^O_p_ values vary by TELMs with larger circles and bars indicating mean ±1σ per TELM (points have been horizontally jittered slightly for visual clarity). The green and blue shaded rectangles indicate the seasonal range of δ^18^O_p_ * values for the 3× and 6× pre-industrial CO2 levels predicted by the iCESM simulation integrated from surface to 25 m (Zhu et al., 2020).

In contrast to bivalves, the bulk δ^18^O_p_ values from elasmobranchs exhibit greater variability than expected considering predicted seasonal δ^18^O_p_* values from the Zhu et al. (2020) iCESM simulations (Figure 4, Table 2, Table SI4.1 in Supporting Information S1). Model δ^18^O_p_* values predict the seasonal variation at Seymour to range from 20.0 to 22.3%o for the 3× pre- industrial CO_2_ case and 19.0–21.2%o for the 6× case during the Eocene (Figure 4, Table SI4.1 in Supporting Information S1). Between the two scenarios, the 3× pre-industrial CO_2_ level simulation captures variability closer to that observed in elasmobranchs, but the variation in empirical δ^18^O_p_ values exceeds the seasonal range derived from the model predictions (Figure 4). The largest discrepancy between mean δ^18^O_p_ values across TELMs and model predictions is during TELM 2 and 6 (Figure 4, Table 1) where a large number of observations are greater than 22.1%o (Figure 4, Table 2, Table SI4.1 in Supporting Information S1). In addition, several outliers are observed across TELMs, often with values between 16.6 and 18.7%o, showing a *z* score lower than - 2 (Figure 4; Table SI7.1 in Supporting Information S1). FTIR spectra suggest that these outliers are not a result of diagenetic alteration (Supporting Information SI2 in Supporting Information S1).

### 5.4 Spatial δ^18^O_p_* Estimates From Model Simulations

**Figure 5:**
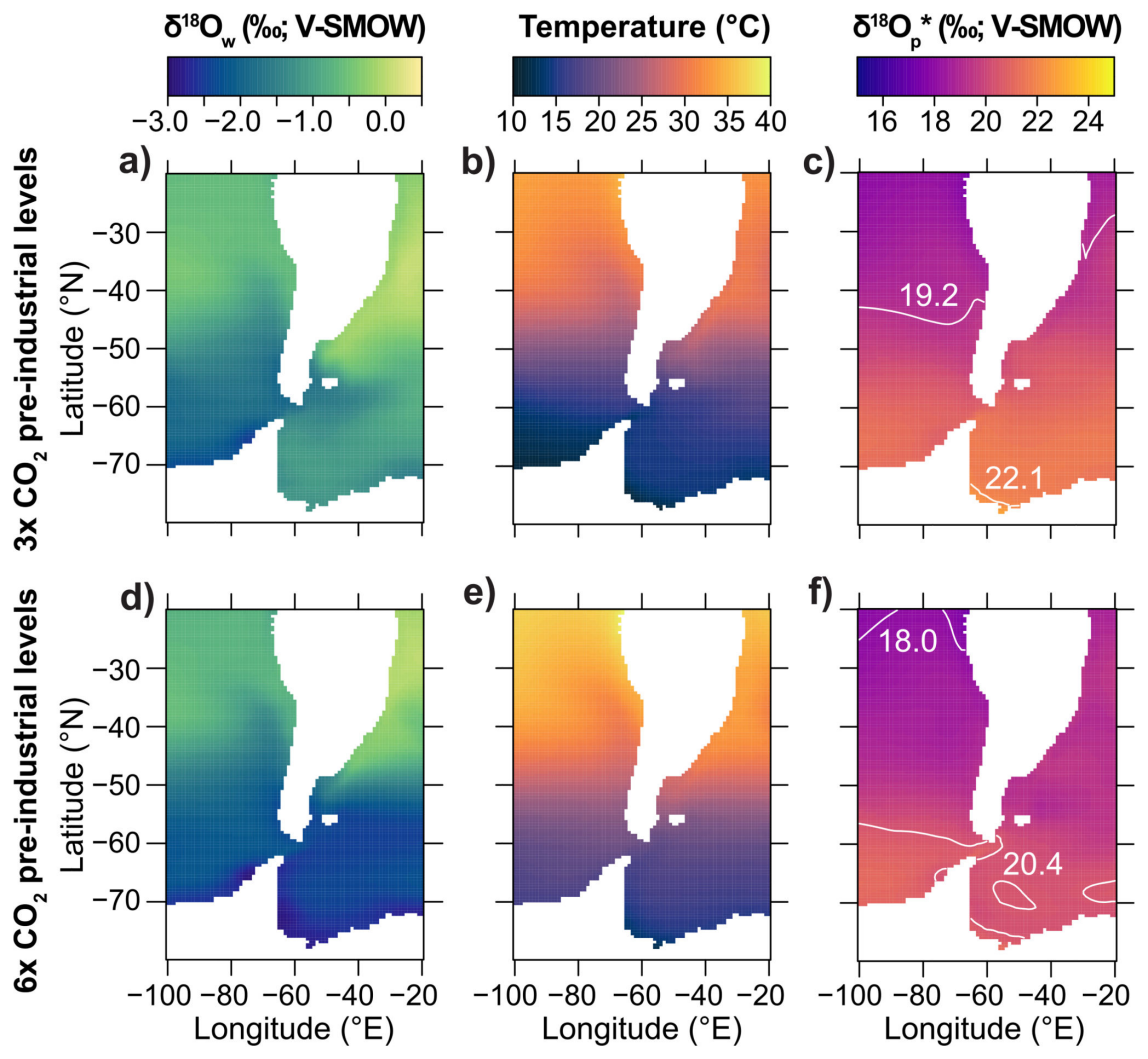
Early Eocene simulations from Zhu et al. (2020) model sea surface temperature (b and e) and δ^18^O_w_ values (a and c) across a spatial distribution within 25 m b. s.l. Arrays of these environmental conditions under 3× (first row) and 6× (second row) pre-industrial CO_2_ levels were used to predict δ^18^O_p_* values (c and f) using Equation 2 (Lécuyer et al., 2013) to compare to empirical values from shark enameloid. The simulated δ^18^O_p_* values for both simulations report the mean ±2σ isoscape values in white to define the spatial variation captured by simulations.

We use sea surface temperature and δ^18^O_w_ values from iCESM output (Zhu et al., 2020) to estimate δ^18^O_p_* values in the region between the South America and Antarctic continents (Figure 5). Spatial δ^18^O_p_* distribution for the 6× pre-industrial CO_2_ levels exhibit δ^18^O_p_* mean ± 2σ values of 18.0 and 20.4%o, which are too low compared to values in elasmobranch teeth and are observed only as outliers (Figure 5). In contrast, model δ^18^O_p_* distribution for the 6× pre-industrial CO_2_ levels captures the variation from empirical measurements of elasmobranch bioapatite δ^18^O_p_ values with mean ± 2σ values of 19.2 and 22.1%o across the latitudinal gradient in both Pacific and Atlantic sectors (Figure 5). However, δ^18^O_p_ values from elasmobranch bioapatite exhibit larger variations, with values being up to ~2.0%o higher than those predicted from model simulation for the 3× CO_2_ case (Figure 4, Table 2). This suggests that the simulated spatial δ^18^O_p_* distribution within shallow waters cannot fully explain the variation observed in elasmobranch bioapatite.

### 5.5 Environmental Conditions Experienced by Pelagic and Benthic Sharks Across TELMs

**Figure 6:**
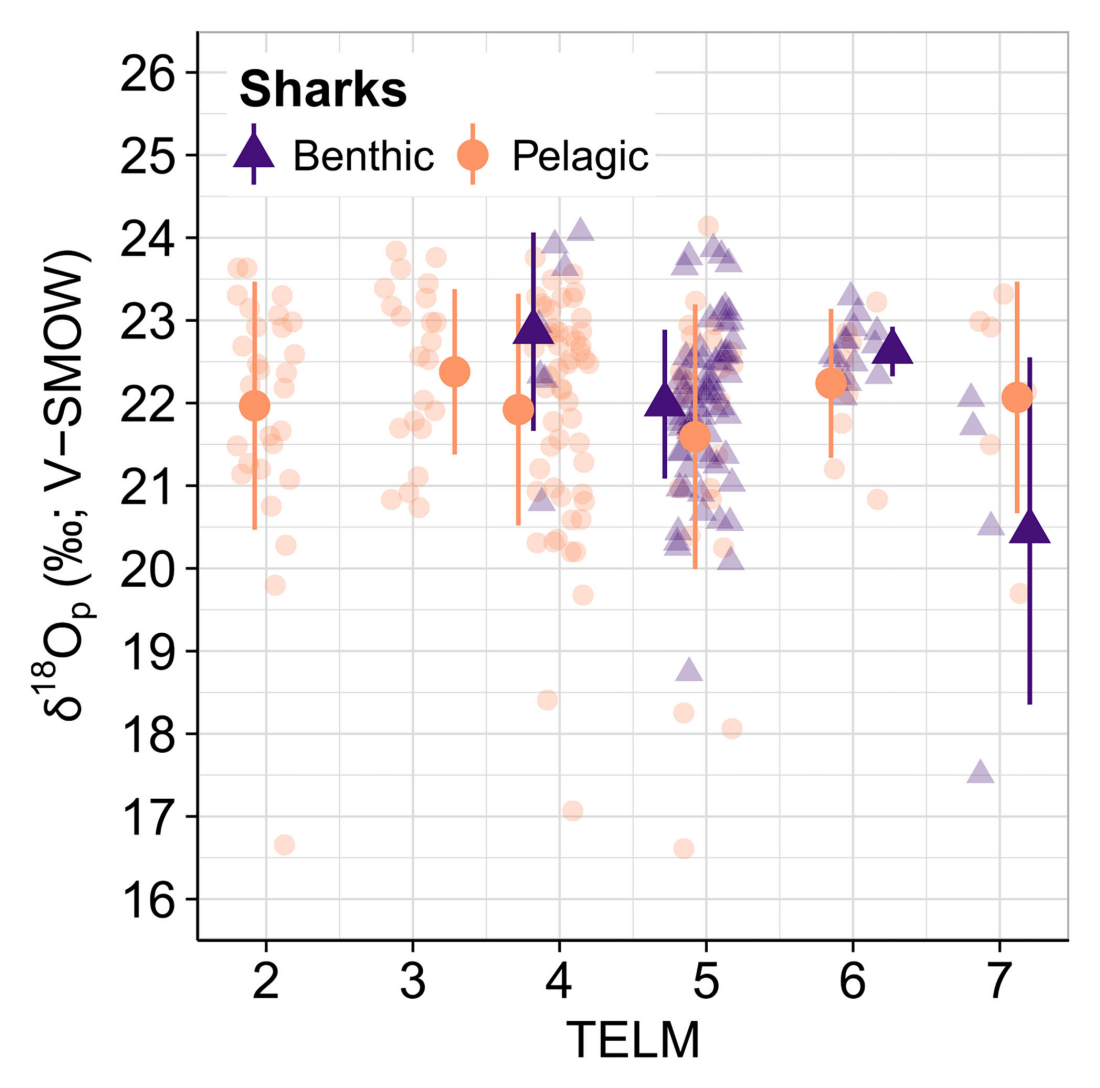
Pelagic (orange circles) and benthic (violet diamonds) elasmobranchs from LMF and SMF have similar δ^18^O_P_ values that suggest they lived in very similar environments across TELMs. Shaded points are individual observations, while solid points and bars are mean ±1σ values of groups across TELMs (points have been horizontally jittered slightly for visual clarity).

Our data set comprises elasmobranch tooth specimens from pelagic (*n* = 149; δ^18^O_p_ = 22.0 ± 1.4%o) and benthic (*n* = 94, δ^18^O_p_ = 22.1 ± 1.1%o) taxa with δ^18^O_p_ values similar between groups (Figure 6, Table 4). Mean δ^18^O_p_ values from pelagic specimens dropped from 22.0 ± 1.5%o in TELM 2 to a minimum of 21.6 ± 1.6%o in TELM 5, then increased to a maximum of 22.2 ± 0.9%o in TELM 6 (Figure 3a, Table 4). Benthic taxa follow a similar trend with slightly higher δ^18^O_p_ values compared to their pelagic relatives (Figure 6, Table 4), although this distinction is not statistically significant based on a Kruskal-Wallis test (Table 4).

## 6 Discussion

### 6.1 No Trend in Elasmobranch Bioapatite δ^18^O_p_ Variation Across the Eocene

The observed mean and median in bulk δ^18^O_p_ values of elasmobranch teeth from the Middle and Late Eocene deposits of LMF and SMF do not show significant shifts over time (Figure 2, Tables 2 and 3), which at face value suggests no directional environmental change through the Eocene in this setting. These results are unexpected given evidence for global paleoceanographic and paleoclimatic trends as well as marked changes in the compositions of marine bivalves in the section (Ivany et al., 2008). One possible explanation for this trend across TELM units could be a time-averaging effect within individual teeth, reflecting environmental signals over the period during which the teeth form. The temporal resolution available at LMF and SMF are over millions of years per TELM while the δ^18^O_p_ values of elasmobranchs indicate snapshots of environmental conditions during mineralization. If, for example, teeth are consistently produced in one season over another, the lack of a trend over time could mean that δ^18^O_p_ values of elasmobranch bioapatite do not record the full range of environmental conditions experienced in LMF and SMF waters. However, this mismatch in temporal resolution is not an issue for co-occurring bivalves. Bulk δ^18^O_c_ values from *Cucullaea* and *Retrotapes* exhibit a shift toward higher values across TELMs (*Cucullaea* mean δ^18^O_c_ values shift from 0.02 ± 0.34%o in TELM 2 to 1.04 ± 0.43%o in TELM 7; *Retrotapes* mean δ^18^O_c_ values shift from 0.72 ± 0.18%o in TELM 2 to 0.90 ± 0.32%o in TELM 7) (Ivany et al., 2008) (Figure SI5.1 and Table SI5.1 in Supporting Information S1). These two bivalve species record environmental conditions in their carbonate oxygen isotopes with alternate growing seasons consistent with the seasonal variability captured by the forward iCESM simulation at 3× pre-industrial CO_2_ levels, with *Cucullaea* usually biased toward the colder, winter temperatures (i.e., ^18^O-enriched values) than *Retrotapes* (Ivany et al., 2008; Judd et al., 2019) (Figure 3, Figure SI5.1, and Table SI5.1 in Supporting Information S1). Despite this slight seasonal bias, δ^18^O_c_ values are higher toward the top of the sedimentary succession, especially in TELM 7 where δ^18^O_c_ values of both taxa exceed the seasonal δ^18^O_c_* range for Seymour Island as predicted by the iCESM simulation (Figure SI5.1 and Table SI5.1 in Supporting Information S1). The discrepancy between modeled and bivalves δ^18^O_c_ values in TELM 7 could arise from the iCESM simulation’s boundary conditions being scaled for the Early Eocene (Zhu et al., 2020), resulting in less correspondence between later TELM predictions and empirical results. Overall, bulk δ^18^O_c_ values of bivalves increase toward the Late Eocene, consistent with global marine records of deep-sea foraminifera δ^18^O values that reflect a combination of cooling temperatures and higher δ^18^O_w_ values, the latter of which is influenced globally by changes in continental ice volume and other regional factors (Zachos et al., 2001). While isotopic composition in elasmobranch bioapatite tracks averaged environmental signals as teeth mineralize (Vennemann et al., 2001), time-averaging effects cannot explain the lack of trends across TELM units in δ^18^O_p_ values of elasmobranchs.

Similarly, time-averaging effects that occurred during the deposition of TELMs cannot explain the environmental stasis observed in elasmobranch’ δ^18^O_p_ values across time. Some sediment reworking effects were hypothesized in TELM 4 given that a significant number of co-occurring bivalve shells have a carbon and oxygen isotopic composition similar to those recovered from TELM 3 (Ivany et al., 2008; Figure SI5.1 in Supporting Information S1). However, multiple pieces of evidence exclude strong sediment reworking effects: (a) palynological analysis of TELMs reveals distinct assemblages (Aménabar et al., 2020): (b) empirical and simulated seasonally resolved precipitation trends in Seymour Island indicate little difference in precipitation regimes between winter and summer seasons (Judd et al., 2019).

Nor is diagenesis likely to offer an explanation: TELM deposits were not buried below 1 Km and did not experience temperatures higher than 80°C (Marenssi et al., 2002); and diagenetic recrystallization of co-occurring bivalves *Cucullaea* and *Retrotapes* has been extensively studied in the past. Shells usually display primary shell microtextures, an aragonite mineralogy, and comparable trace element concentrations to modern bivalves (Buick & Ivany, 2004; Douglas et al., 2014; Dutton et al., 2002; Ivany et al., 2008; Judd et al., 2019). Moreover, FTIR analysis suggests that diagenetic alteration in elasmobranch bioapatite materials is minimal (Sections 3.2.1 and 3.2.2; Supporting Information SI2 in Supporting Information S1).

Other possible explanations for the lack of observed environmental trends across TELMs could derive from analytical uncertainty. The variability in elasmobranch bioapatite δ^18^O_p_ values is larger compared to forward simulations, with 1σ ranging from 0.6 to 1.8%o within TELMs when considering bulk measurements (Figure 2, Table 2). However, analytical uncertainty as 1σ is low (≤0.4%o) when monitoring reference materials. Therefore, variability in δ^18^O_p_ values of elasmobranch bioapatite reflects a real signal of habitat heterogeneity captured during sharks’ and rays’ lifetimes due to some combination of environments experienced in the Seymour Island setting and other places to which the animals may have traveled.

### 6.2 Discerning Migration and Sedentary Habits From δ^18^O_p_ Values of Pelagic and Benthic Elasmobranch Species

The lack of observed environmental trends across TELMs from elasmobranch bioapatite δ^18^O_p_ values could derive from seasonal and ontogenetic movement patterns. Environmental conditions such as temperature and salinity combined with prey availability are primary drivers that influence movements of elasmobranchs. Some elasmobranchs undergo extensive latitudinal and vertical movements to track their preferred temperatures, salinity, and biological conditions across horizontal or vertical isoclines, while others display more sedentary habits (Compagno, 2002; Kneebone et al., 2012; Schlaff et al., 2014). The distribution of δ^18^O_p_ values from elasmobranch teeth partially aligns with seasonal, local trends at Seymour Island but also suggests a spatial extension based on δ^18^O_p_* values from the forward 3× CO_2_ iCESM simulation (Figures 4 and 5). Given the combination of variable lifestyles and the time elapsed between tooth mineralization and shedding in different shark and ray species, the δ^18^O_p_ variability of elasmobranch bioapatite collected from LMF and SMF likely record seasonal signals at Seymour Island and the surrounding region.

We developed a framework to hypothesize habitat use among the most abundant pelagic and benthic taxa in our data set across TELMs (i.e., taxa with *n* ≥ 4, see also Table 1). We compared δ^18^O_p_ distribution from elasmobranch bioapatite with co-occurring bivalves after applying transfer Functions 3 and 4 (Table SI5.2 in Supporting Information S1; Ivany et al., 2008; Kim et al., 2015; Longinelli & Nuti, 1973b) and predicted δ^18^O_p_* values based on iCESM simulations for 3× pre-industrial CO_2_ level scenario using Equation 2 (Lécuyer et al., 2013). We extracted seasonal δ^18^O_p_* range for Seymour Island and southern Chile (Supporting Information SI6 in Supporting Information S1), an area within migration range of elasmobranchs that includes coeval fossil assemblages from the Río Turbo and Loreto Formations (Magallanes Basin) with a similar diversity of elasmobranch taxa to

**Figure 7:**
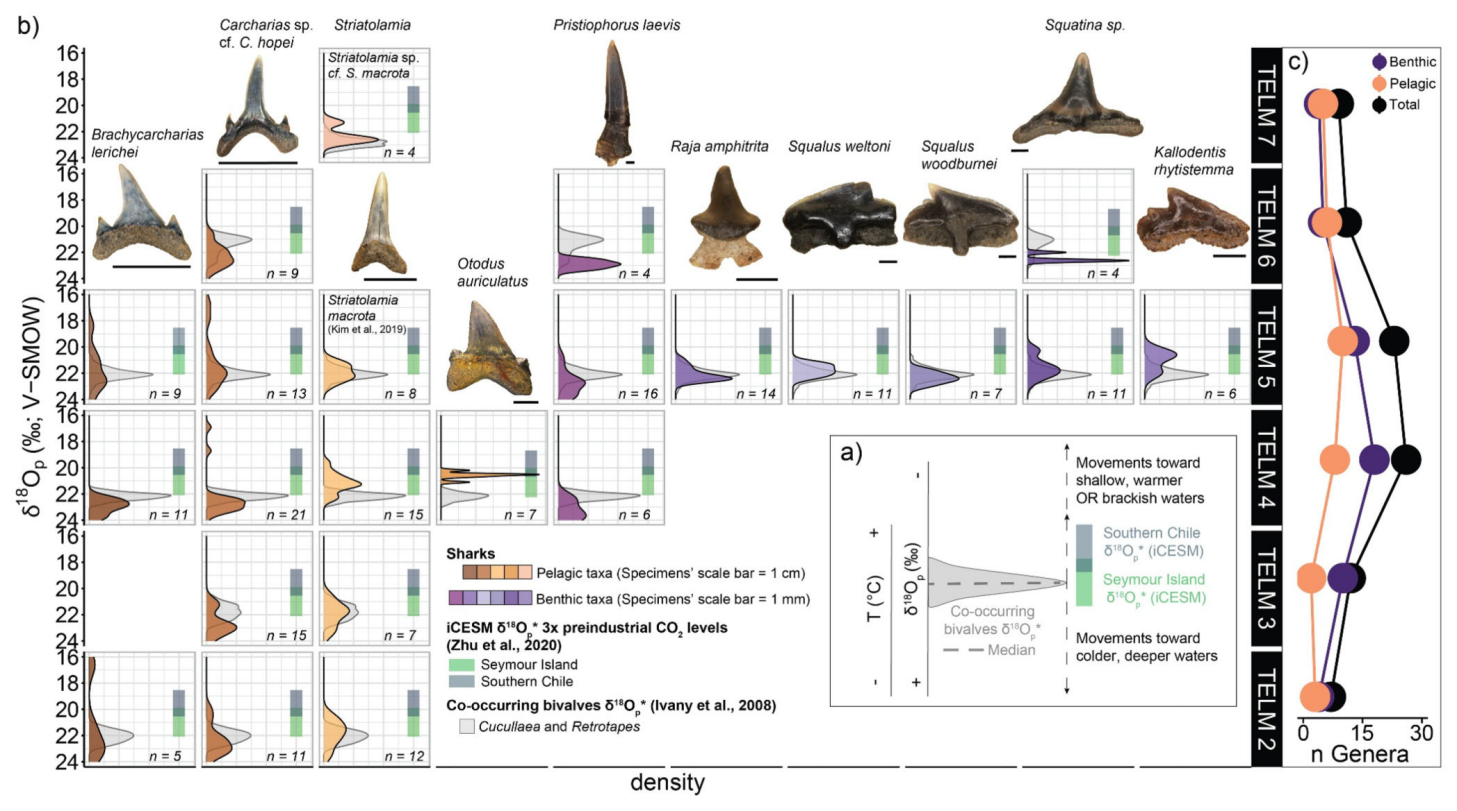
Deciphering the habitat use of elasmobranchs offers context to understand the rise and fall in diversity across TELMs. (a) A schematic representation of the framework adopted to investigate sharks’ habitat use. Co-occurring bivalves’ δ^18^O_p_* distribution is in gray, with the dashed line representing the median. Model seasonal δ^18^O_p_* values for southern Chile and Seymour Island are represented as dark gray and green boxes, respectively. Dashed arrows indicate shifts toward lower (i.e., warmer, shallow waters or brackish environments) or higher δ^18^O_p_ values (i.e., colder, deeper waters) than predicted δ^18^O_p_*. (b) Comparison between δ^18^O_p_ values of pelagic and benthic taxa with transposed values from co-occurring bivalves and δ^18^O_p_* values based on iCESM simulations reveals potential habitat use of different species. The density distribution of shark taxa (columns) across different TELM units (rows). The figure features representative tooth specimens of taxa used for stable isotope analysis. All specimens are depicted in labial view, except for the *S. macrota* specimen, which is shown in lingual view. The scale bar represents 1 cm for pelagic taxa and 1 mm for benthic species. (c) Number of chondrichthyan genera (i.e., sharks, rays, and chimeras) trends across TELM units. The list of taxa was compiled from the literature (Engelbrecht et al., 2016a, 2016b, 2017a, 2017b, 2019; Kriwet, 2005; Kriwet et al., 2016; Long, 1992b; Marramá et al., 2018). Colors indicate trends for the total number of chondrichthyan genera (black), benthic (purple), or pelagic taxa (orange) per TELM unit.

**Figure 8:**
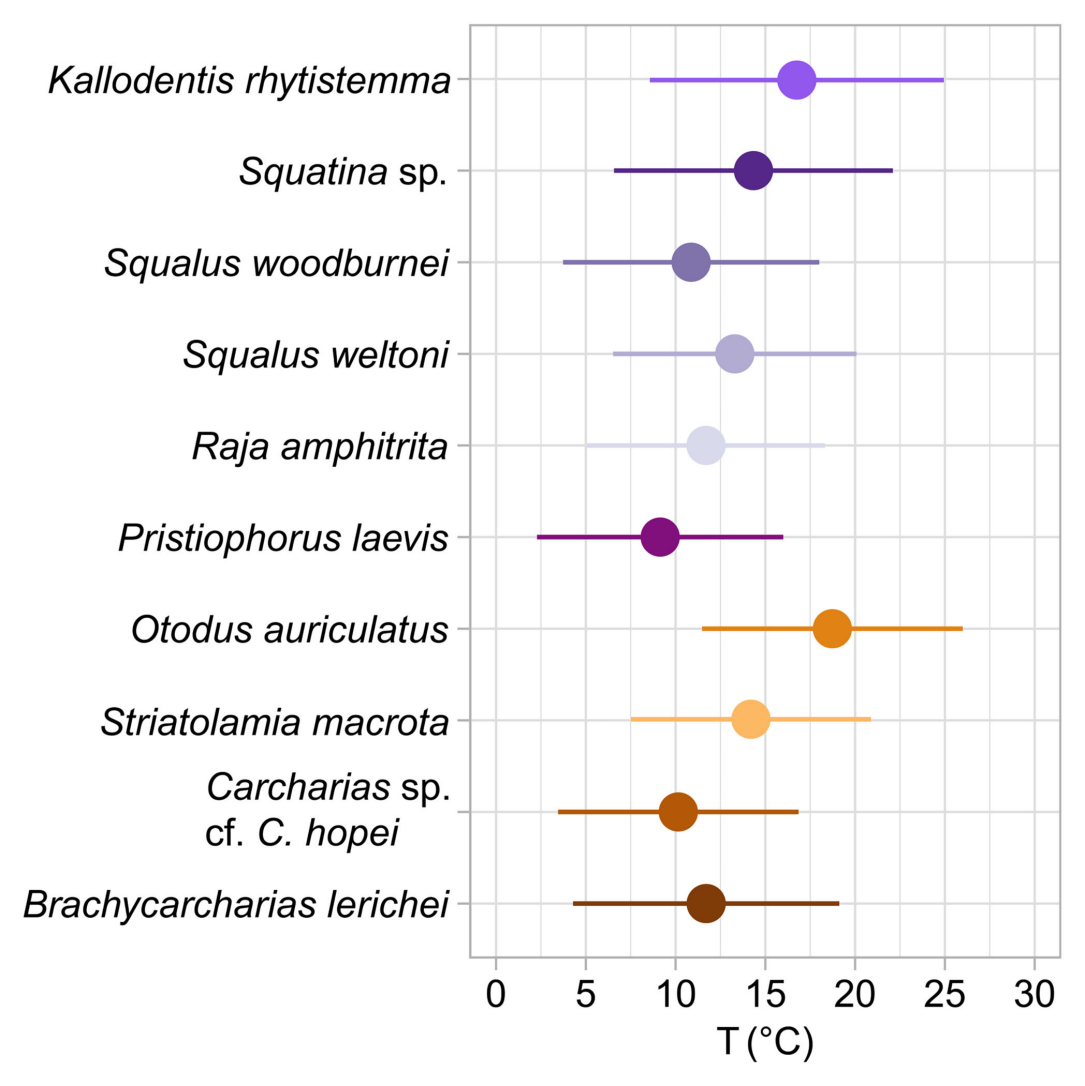
Temperature estimates from δ^18^O_p_ measurements of pelagic (brown-yellow color palette) and benthic taxa (purple-gray color palette) using the Bayesian framework explained in Supporting Information SI2 in Supporting Information S1. Dots indicate mean temperature values, while bars are 95% confidence intervals. The simulation uses prior model seawater temperature and δ^18^O_w_ with a range of −0.43–35.58°C and −2.85–0.00%o, respectively (Supplementary Information SI3 in Supporting Information S1).

Seymour Island (Otero & Soto-Acuña, 2015a). Species that exhibit distributions in agreement with the predicted values for Seymour Island and co-occurring bivalves indicate local, seasonal conditions resulting from a relatively sedentary habit without long-range migration (Figure 7a). Elasmobranch taxa that exhibit a broader variation in δ^18^O_p_ values or shifts in their median value compared to δ^18^O_p_* values of bivalves and those predicted from the model simulation in Seymour Island are likely capturing environmental conditions over a broader region, including warmer, low latitudes or deeper, colder depths, or brackish environments with low δ^18^O_w_ values (Figure 7a). We estimated preferred temperatures of elasmobranch taxa using a Bayesian approach that considers model temperature and δ^18^O_w_ outputs for the 3× CO_2_ between Antarctica and South America case as priors (Griffiths et al., 2023; Zhu et al., 2020; full description in Supporting Information SI2 in Supporting Information S1). Temperature values (T(°C)) are reported as mean ±95% confidence interval (CI; Figure 8, Table SI8.1 in Supporting Information S1).

Pelagic and benthic species on the whole have similar mean δ^18^O_p_ values, but their δ^18^O_p_ distributions indicate differences in preferred environments across TELMs (Figure 7b). Compared to predicted δ^18^O_p_* values, the distribution of δ^18^O_p_ values of pelagic taxa indicates movement toward warmer, lower-latitude settings like southern Chile or to brackish environments (Figure 7b; Tables SI5.2, SI6.1, and SI7.1 in Supporting Information S1). For example, the sand tiger sharks *Brachycarcharias lerichei, Carcharias* sp. cf. *Carcharias hopei*, and *Striatolamia* (including *S. macrota* and *Striatolamia* sp. cf. *S. macrota*) exhibit a broad variation in δ^18^O_p_ values, overlapping with transposed δ^18^O_p_ values of bivalves and δ^18^O_p_* predictions from model outputs for Seymour Island in some TELMs (i.e., TELM 2 and TELM 3 for *B. lerichei* and *Carcharias* sp., TELM 2 to 4 for *S. macrota*, and TELM 7 for *Striatolamia* sp. cf. *S. macrota*; Figure 7b, Tables SI5.2, SI6.1, and SI7.2 in Supporting Information S1). Among the sand tiger sharks, *Striatolamia* has lower mean and median values compared to other pelagic taxa, suggesting a preference for shallower and/or warmer waters compared to *B. lerichei* and *Carcharias* sp. (Figure 7b; Tables SI5.2, SI6.1, and SI7.2 in Supporting Information S1). Temperature estimates (i.e., T(°C) ± CI) agree with this interpretation, showing that *S. macrota* preferred temperatures of 14.2 ± 7.4°C in contrast with *B. lerichei* and *Carcharias* sp., which preferred temperatures of 11.7 ± 7.4°C and 10.2 ± 6.4°C (Figure 8, Table SI8.1 in Supporting Information S1). Finally, *B. lerichei* and *Carcharias* sp. from TELM 4 and 5 have several more ^18^O-depleted outliers, lower than any predicted δ^18^O_p_* values (Figures 4, 7b, and 8, Table SI7.1 in Supporting Information S1). These outliers could indicate movements toward the inner part of the estuary in Seymour Island (Marenssi et al., 2002; Porębski, 2000), a brackish environment with meteoric input influence, resulting in lower δ^18^O_w_ and δ^18^O_p_ values at the time of tooth formation.

Extant sand tiger sharks migrate seasonally to maintain suitable temperature and salinity conditions (Kneebone et al., 2014), indicating that their ability to track isoclines in space and time could be a conservative trait in their lineages. For example, δ^18^O_p_ values of the fossil sand tiger sharks suggest that they tracked preferred temperatures across latitudes similar to their modern analog *C. taurus* (Kim et al., 2014, 2020), indicating that this trait is conservative in their lineages. In addition, movements to estuarine environments could be another conservative trait among sand tiger sharks. Prolonged residency in such habitat, especially in juvenile individuals, is documented for both modern *C. taurus* (e.g., in Plymouth, Kingston, Duxbury Bay, USA) and fossil *S. macrota* and *Carcharias* cf. *cuspidatus* specimens from the Arctic (e.g., Eureka Sound, Canada) and the Alps (e.g., Swiss Molasse Basin, Switzerland) (Kim et al., 2014, 2020; Kneebone et al., 2012; Kocsis et al., 2007).

Among the pelagic taxa, *Otodus auriculatus* has a δ^18^O_p_ distribution that does not overlap with bivalves or simulated δ^18^O_p_* (Figure 7b, Table SI7.2 in Supporting Information S1). This taxon exhibits a 2%o shift in median δ^18^O_p_ values from that of bivalves’ equivalent δ^18^O_p_ (*O. auriculatus* median δ^18^O_p_ = 20.5%o, TELM 4 Bivalve median δ^18^O_p_* = 22.2%o), with values falling in between predicted seasonal δ^18^O_p_* range for Seymour Island and Chile (Figure 7b, Tables SI5.2, SI6.1, and SI7.2 in Supporting Information S1). Interestingly, *O. auriculatus* is the taxon with the narrowest variation in δ^18^O_p_ values, which are also the lowest compared to other elasmobranch species (Figure 7b, Table SI7.2 in Supporting Information S1). This results in warmer temperature preferences of 18.7 7.3°C compared to other fossil shark taxa, which aligns with the preferred range of the modern *C. carcharias* (Figure 8, Table 1, and Table SI8.1 in Supporting Information S1; Bradford et al., 2020; Froese & Pauly, 2024). We assumed an ectotherm physiology for all elasmobranchs in this study with the assumption that δ^18^O_p_ values tracked temperature and δ^18^O_w_ isoclines. However, the relatively low and consistent δ^18^O_p_ values could indicate the ability of *O. auriculatus* to maintain a constant body temperature warmer than the surrounding water.

Endothermy is common in lamniform sharks, including extant species (e.g., *C. carcharias*) and recent isotope results from the *Otodus* lineage (Griffiths et al., 2023; Watanabe et al., 2019), which could indicate an evolutionary history of endothermy within this lineage. With endothermy, δ^18^O_p_ values would reflect consistent elevated temperatures with variation primarily due to δ^18^O_w_ gradients with salinity, depth, or latitude. Combined δ^18^O_p_ and clumped isotope measurements (i.e., Δ_47_) on the same tooth specimens could support this hypothesis.

Benthic taxa in our data set display species-specific variability in habitat use (Figure 7b, Table SI7.2 in Supporting Information S1). The δ^18^O_p_ distributions of *Raja amphitrita, Squalus weltoni*, and *Squalus woodburnei* are in agreement with δ^18^O_p_* of co-occurring bivalves and δ^18^O_p_* range of Seymour Island from the iCESM model (Figure 7b; Tables SI5.2, SI6.1, and SI7.2 in Supporting Information S1). Temperature estimates for these taxa are 11.7 ± 6.6°C, 13.3 ±6.8°C, and 10.9 ±7.1°C for *R. amphitrita, S. weltoni*, and *S. woodburnei*, respectively (Figure 8, Table SI8.1 in Supporting Information S1), which suggest that these species inhabited coastal shelf habitats emblematic of the La Meseta Formation, and their bioapatite tracks local, seasonal environmental conditions. A comparatively sedentary habit for these species is in contrast to the active lifestyles of their modern analogs *Bathyraja griseocauda* and *Squalus acanthias*, which frequently move between coastal areas and the seafloor of the continental slope (Arkhipkin et al., 2012; Casselberry & Carlson, 2015; Engelbrecht et al., 2017a, 2019; Sulikowski et al., 2010). In contrast, the saw shark *Pristiophorus laevis* has median δ^18^O_p_ values consistently higher than co-occurring bivalves and predicted δ^18^O_p_* values (Figure 7b; Tables SI5.2, SI6.1, and SI7.2 in Supporting Information S1), suggesting a preference for colder and deeper environments (T(°C) ± CI =9.1 ±6.9°C; Figure 8, Table SI8.1 in Supporting Information S1). This behavior is common in the modern *Pristiophorus cirratus*, a taxon occurring on the continental shelf at depths between 40 and 630 m b. s.l. (Froese & Pauly, 2024; Raoult et al., 2020). Finally, δ^18^O_p_ values of *Squatina* sp. and *Kallodentis rhytistemma* in TELM 5 exhibit a bimodal distribution, with modes overlapping with predicted δ^18^O_p_* values of co-occurring bivalves and with those estimated for southern Chile and Seymour Island from the iCESM model (Figure 7b). The sample size for *Squatina* sp. in TELM 6 is too small for reliable inferences but the δ^18^O_p_ values of *Squatina* sp. and *K. rhytistemma* from TELM 5 indicate that these taxa were active species inhabiting shallow, warm waters with estimated temperature preferences of 14.3 ± 7.8°C and 16.8 ± 8.2°C, respectively (Figure 8, Table SI8.1 in Supporting Information S1). These findings contrast with habitat preferences of their modern analogs *Squatina squatina* for *Squatina* sp. and *T. semifasciata* for *K. rhytistemma*, which themselves differ from one another: *T. semifasciata* is an active bottom-dweller in estuarine environments with seasonal movement patterns, while *S. squatina* is a sedentary taxon inhabiting coastal settings (Carlisle & Starr, 2009; Meyers et al., 2017; Nosal et al., 2014). Instead, *K. rhytistemma* and *Squatina* sp. likely had habitat preferences similar to their co-occurring pelagic taxa, favoring shallow, warmer waters and exhibiting seasonal migration to lower latitudes (Figures 7b and 8; Tables SI7.2 and SI8.1 in Supporting Information S1).

In summary, the inferred habitat use strategies suggest that δ^18^O_p_ values of different elasmobranch taxa can reflect local environmental signals at Seymour Island or regional signals along spatial gradients. Of note, transposed δ^18^O_p_ values of bivalves and empirical δ^18^O_p_ measurements from *Striatolamia* sp. cf. *S. macrota* teeth poorly overlap with seasonal simulated values for Seymour Island. Again, the mismatch is likely driven by boundary conditions unsuitable for capturing seawater temperature and δ^18^O_w_ values for the Late Eocene. Although we cannot precisely determine the paths of migratory elasmobranchs, isotope-enabled climate simulations provide predictions to track environmental conditions.

### 6.3 Do δ^18^O_p_ Values of Elasmobranchs Support Regional or Global Environmental Changes?

We explored evidence for stronger thermal stratification between warm shallow and cold deep waters by comparing δ^18^O_p_ values of pelagic and benthic elasmobranchs within and between TELMs (Figure 6, Table 4). This approach is analogous to the Mg/Ca ratios observed in planktic and benthic foraminifera from Eocene-Oligocene deposits at Maud Rise (Southern Atlantic Ocean, ODP 689b), where benthic foraminifera encountered colder conditions as the Drake Passage deepened (Hodel et al., 2021). We expected that benthic elasmobranchs would have higher mean δ^18^O_p_ values than their pelagic relatives in the younger TELMs, suggesting movements off the shelf to deeper, colder environments if the Drake Passage had deepened (Arkhipkin et al., 2012; Casselberry & Carlson, 2015; Engelbrecht et al., 2017a; Hodel et al., 2021; Lagabrielle et al., 2009; Marenssi et al., 2002; Montes et al., 2013; Sulikowski et al., 2010). Contrary to our expectations, we found no statistically significant difference between the two groups (Table 4); pelagic and benthic taxa from Seymour Island indicate the presence of a similar suite of environmental conditions across all TELMs (Figures 5 and 7b, Table 4, Table SI7.2 in Supporting Information S1). This result suggests either that sharks never migrated off the shelf to experience any cooling of deep waters, or that there was only weak thermal stratification and thus no deepening in the Drake Passage area during sedimentation of LMF and SMF.

One possibility is that δ^18^O_p_ values of pelagic and benthic elasmobranchs reflect differences not only in temperature but also in salinity. While δ^18^O_p_ values for the two groups are indistinguishable, it is conceivable that each group experienced a different combination of temperature and salinity such that a decrease in salinity effectively canceled out the δ^18^O_p_ signal of any temperature decrease over time. Mineralogical and geochemical evidence in the Atlantic sector of the Southern Ocean shows a discrete amount of continental input in the seawater as ice caps expanded and melted, which were redistributed by the westward Antarctic Coastal Current along the Weddell Sea and Antarctic Peninsula (Carter et al., 2017; Scher et al., 2011, 2014). The coast along the Weddell Sea would be influenced by these cold freshening events, which would affect the δ^18^O_p_ values in elasmobranchs.

However, it is unlikely elasmobranchs would track regional changes in salinity so closely. First, this regional freshening is constrained to nearshore, coastal waters and primarily affects the isotopic composition of elasmobranchs inhabiting shallow waters in Seymour Island—mostly pelagic and some benthic taxa. If major regional salinity shifts had occurred, we would expect lower mean and median δ^18^O_p_ values in taxa living close to the surface and nearshore in younger TELMs compared to simulated values and co-occurring bivalves, or between the same elasmobranch taxa relative to older TELMs, which is not the case. Local recorders like *R. amphitrita, S. weltoni*, and *S. woodburnei* track salinity gradients in good agreement with mollusks and simulated δ^18^O_p_* values, and pelagic taxa like the sand tiger sharks have just a handful of low δ^18^O_p_ values (Figures 4 and 7b, Supplementary Information SI7 in Supporting Information S1). The latter result indicates that sand tiger sharks could live in or move to brackish waters during the early stages of ontogenetic developments (Kim et al., 2022) rather than reflecting regional salinity changes. Today, adult sand tiger sharks move seasonally (Kneebone et al., 2014); in the Eocene, these taxa could potentially move to areas where such regional shifts in salinity would not occur (e.g., southern Chile; Figure 7b). Lastly, seasonal freshening due to ice melting minimally lowers seawater salinity by approximately 0.2 PSU in waters above the pycnocline in today’s Weddell Sea (Graham et al., 2013), leading to shifts in δ^18^O_w_ values that are ~0.05%o lower than expected (calculated using the Southern Ocean δ^18^O_w_—salinity relationship from LeGrande & Schmidt, 2006, δ^18^O_w_ = 0.24*salinity (PSU) -8.45). Therefore, it is unlikely that δ^18^O_p_ values of elasmobranchs track these minor salinity changes in seawater.

Another plausible reason for the lack of change between pelagic and benthic taxa mean and median δ^18^O_p_ across TELMs is that elasmobranchs track their preferred temperature and salinity gradients, which shape species-specific niche spaces. This hypothesis better explains why δ^18^O_p_ values of elasmobranchs provide a broad, regional (or more localized if they move in brackish waters) environmental perspective instead of the environmental and oceanographic changes documented in global records. This tracking behavior may also explain the decline in diversity toward the Late Eocene (Figures 7b and 7c), where global changes exerted selective pressure on elasmobranchs, favoring species adapted to colder conditions. Global cooling and oceanographic changes throughout the Eocene could result in increased productivity and intensification of wind-driven upwelling of cold waters in the Weddell Sea (Amenábar et al., 2020; Borrelli et al., 2021; Ladant et al., 2018; Scher & Martin, 2006). These environmental and oceanographic shifts could cause physiological challenges for many elasmobranch taxa, as well as for their prey (Lubitz et al., 2024; Vilmar & Di Santo, 2022). Indeed, such changes likely impacted sharks on Seymour Island, leading to a decline in chondrichthyan diversity from TELM 5 onward (Figure 7c). Among the species we analyzed, only *Carcharias* sp., *P. laevis*, and *Squatina* sp., along with a few other taxa not analyzed here, persisted after TELM 5 (Engelbrecht et al., 2017a, 2017b; Kriwet, 2005; Kriwet et al., 2016). Among these three species, we were perplexed that *P. laevis* is the only benthic taxon evidently preferring colder, potentially deeper waters, and such habitats are available to *Carcharias* sp. across all TELMs (Figures 7b and 8, and Table SI7.2 in Supporting Information S1). These isotopic and occurrence data suggest that some elasmobranch species exhibit ecological plasticity and can withstand environmental changes. For example, *S. macrota* occurs in LMF deposits only, from TELM 1 to 5 (Lutetian to Bartonian age, Middle to Late Eocene) and exhibits similar mean δ^18^O_p_ but a decreasing range of variation through time (1σ decreases from 1 to 0.6%o) (Figure 7b, Table SI7.2 in Supporting Information S1; Kriwet et al., 2016; Long, 1992b; Kim et al., 2020). The falling variation in δ^18^O_p_ values combined with occurrence data indicate that *S. macrota* reduced its geographic range as high-latitude waters turned colder until cooling and associated changes drove the extirpation of this taxon (Kim et al., 2020). Global cooling and associated environmental change could also explain the worldwide decline of *S. macrota* in other settings at varying latitudes, such as the North Sea or the Eocene deposits of Southern Chile at the beginning of the Late Eocene (i.e., Bartonian) (Cappetta, 2012; Kim et al., 2020; Otero & Soto-Acuña, 2015a; Otero et al., 2013; Zachos et al., 2001; Zacke et al., 2009). In contrast, *Carcharias* sp. and *P. laevis* could accommodate Eocene climate cooling given their tolerance to colder conditions. Therefore, the δ^18^O_p_ values in the bioapatite of these taxa may not directly track the environmental changes we anticipated as they preferred similar environmental conditions.

## 7 Conclusions

Geochemistry of elasmobranch teeth records the environmental conditions where individuals lived and offers context on how sharks and rays coped during periods of climate change. Variability in δ^18^O_p_ measurements of elasmobranch tooth specimens from the LMF and SMF shed light on habitat use and regional environmental conditions near Seymour Island during the Eocene. The overall stability in bulk δ^18^O_p_ values across TELMs suggests that sharks and rays generally seek habitats with preferred conditions rather than behaving as passive tracers in a constant location like bivalves or foraminifera.

Unlike co-occurring bivalves, the variability in elasmobranch δ^18^O_p_ values aligns only partially with seasonal predictions from the iCESM model simulation for the Eocene. Similarly, spatial gradients of predicted model outputs in shallow waters do not fully explain the variation in shark δ^18^O_p_ values. The higher δ^18^O_p_ values often observed in some elasmobranchs’ bioapatite compared to predicted model values likely reflect movements to deeper, colder waters not captured by the simulation. Conversely, lower δ^18^O_p_ values than those simulated suggest movements to warmer or brackish, estuarine environments not captured in the simulation.

We explored the potential for the Drake Passage during the Late Eocene by comparing δ^18^O_p_ values between pelagic sharks, typically found in warmer, shallower waters, and benthic taxa capable of traveling to colder, deeper depths far from Seymour Island shore. Benthic elasmobranchs did not, by and large, encounter colder conditions relative to pelagic sharks. We did not observe a significant difference in δ^18^O_p_ values between the groups, suggesting that all lived in the same well-mixed water mass or that individual taxon-specific preferences within each group canceled each other out. Global cooling in the Eocene, though, likely exerted a selective pressure on sharks with those tolerant of colder waters coping better than those with preference for warm water.

Using a framework that combines δ^18^O_p_ values of elasmobranchs with other empirical oxygen isotope measurements and model outputs, we found that some pelagic species evidently traveled to warmer or more brackish settings while others preferred forays into deeper or colder waters. Likewise, benthic species display differing thermal preferences, such that taxon-specific δ^18^O_p_ values say more about the habitat tolerances of mobile taxa than about the environmental conditions at the site in which their teeth are found. These data not only provide insights into the environmental plasticity of elasmobranchs in a changing world, but also offer a valuable framework for refining model simulations of past oceanic conditions, particularly for deeper waters, which remain poorly understood.

## Supplementary Material

Supporting Information may be found in the online version of this article.

S1

S2

S3

S4

S5

S6

S7

S8

S9

S10

S11

S12

S13

S14

S15

S16

S17

Supporting Information

## Figures and Tables

**Table 1:** List of Elasmobranch Taxa and Number of Tooth Specimens per TELM

Taxon	*n* specimens per TELM						Modern analog	Habitat*	T (°C)*	Depth (m b.s.l.)*
	2	3	4	5	6	7				
*Abdounia mesetae*	–	–	1	–	–	–	*Triaenodon obesus* ^l^	benthic	21.1–30.0^a^	0–330^b^
*Brachycarcharias lerichei*	5	–	11	9	–	–	*Carcharias taurus* ^m^	pelagic	9.0–26.9^c^	0–100^c^
*Carcharias* sp. cf. *Carcharias hopei*	11	15	21	13	9	–	*Carcharias taurus* ^n,o^	pelagic	9.0–26.9^c^	0–100^c^
*Carcharocles sokolovi*	–	–	–	–	–	1	*Carcharodon carcharias* ^g^	pelagic	5.0–25.0^d^	0–1,200^e^
Dalatiidae indet.	–	–	–	2	–	–	*Dalatias lichia* ^i^	benthic	2.5–14.3^d^	37–1,794^e^
*Eodalatias austrinalis*	–	–	–	2	–	–	*Dalatias lichia* ^i^	benthic	2.5–14.3^d^	37–1,794^e^
*Kallodentis rhytistemma*	–	–	–	6	–	–	*Triakis semifasciata* ^i^	benthic	12.8–24.0^d^	0–156^e^
*Otodus auricolatus*	–	–	7	–	–	–	*Carcharodon carcharias* ^n^	pelagic	5.0–25.0^d^	0–1,200^e^
*Palaeohypotodus* sp cf. *P.rutoti*	–	–	–	–	–	1	*Odontaspis ferox* ^g^	pelagic	12.2–23.9^d^	10–1,015^e^
*Pristiophorus laevis*	–	–	6	16	4	2	*Pristiophonts cirratus* ^h^	benthic	14.0–17.7^d^	40–630^e^
*Raja amphitrita*	–	–	–	14	1	–	*Bathyraja griseocauda* ^p^	benthic	2.7–7.7^d^	30–1,010^e^
*Squalus* sp	–	–	–	–	1	1	*Squalus acanthias* ^g,i^	benthic	4.2–18.7^d,f^	0–1,978^e,f^
*Squalus weltoni*	–	–	–	11	2	–	*Squalus acanthias* ^g,i^	benthic	4.2–18.7^d,f^	0–1,978^e,f^
*Squalus woodburnei*	–	–	–	7	2	–	*Squalus acanthias* ^g,i^	benthic	4.2–18.7^d,f^	0–1,978^e,f^
*Squatina* sp.	–	–	–	11	4	1	*Squatina squatina* ^g,i^	benthic	7.7–19.4^d^	2–150^e^
*Striatolamia* sp cf. *S.macrota*	–	–	–	–	–	4	*Carcharias taurus* ^g,k^	pelagic	9.0–26.9^c^	0–100^c^
Total *n* specimens	28	22	61	99	23	10				

*Note*. Fossil shark taxa collected from the Eocene deposits of Seymour Island are expected to live in similar habitats, temperature, and depth ranges of their modern analogs. Headers followed by asterisk (*) indicate habitat, temperature, and depth ranges of modern analogs.

a Kaschner et al. (2015).

b Compagno (1984).

c Kneebone et al. (2014).

d Froese and Pauly (2024).

e Weigmann (2016).

f Sulikowski et al. (2010).

g Kriwet et al. (2016).

h Engelbrecht et al. (2016a).

i Engelbrecht et al. (2017a).

j Engelbrecht et al. (2017b).

k Cunningham (2000)

l Parmley et al. (2003).

m Marramá et al. (2018).

n Kriwet (2005).

o Long (1992b).

p Engelbrecht et al. (2019).

**Table 2:** Summary Statistics of Elasmobranchs’ δ^18^O_p_ Values Across TELMs

TELM	*n*	δ^18^O_p_ (V-SMOW)		
		Mean ± 1σ	Median	Range
7	10	21.4 ± 1.8	21.9	17.5–23.6
6	23	22.5 ± 0.6	22.6	20.6–23.4
5	99	21.9 ± 1.2	22.0	16.7–24.0
4	61	22.0 ± 1.4	22.4	16.9–24.0
3	22	22.4 ± 1.0	22.7	20.6–23.8
2	28	22.0 ± 1.5	22.2	16.6–23.7

*Note*. The table shows the lithostratigraphic unit (TELM), the number of specimens per TELM (*n*), and *δ*^18^O_p_ mean, standard deviation (1σ), and range values in V-SMOW scale.

**Table 3:** Pairwise Comparison of Elasmobranchs’ δ^18^O_p_ Values Between TELM Units Performed via post-h8oc Dunn Test

TELM	2	3	4	5	6
3	0.1525	–	–	–	–
4	0.4291	0.1560	–	–	–
5	0.1786	0.0189	0.0720	–	–
6	0.0877	0.3826	0.0820	0.0062*	–
7	0.1716	0.0463	0.1265	0.3232	0.0269

*Note*. Values in the tables are *p* values performed by the test. Values with asterisk (*) indicate statistically significant difference between groups (*p* < 0.05).

**Table 4:** Summary Statistics and Kruskal-Wallis Test of Pelagic and Benthic Elasmobranch’ δ^18^O_p_ Values From TELM 4 to 7

TELM	Habitat	*n*	δ^18^O_p_ (%o V-SMOW)				Kruskal- Wallis test, pelagic versus benthic elasmobranchs	
			Mean ± 1σ	Median	Range		*H* value	*p*
7	pelagic	6	22.1 ± 1.4	22.5	19.7–23.6		2.25	0.13
	benthic	4	20.4 ± 2.1	21.2	17.5–21.9			
6	pelagic	9	22.2 ± 0.9	22.4	20.6–23.4		1.03	0.31
	benthic	14	22.6 ± 0.3	22.6	22.0–23.3			
5	pelagic	30	21.6 ± 1.6	22.0	16.7–24.0		0.51	0.48
	benthic	69	22.0 ± 0.9	22.1	18.5–24.0			
4	pelagic	54	21.9 ± 1.4	22.4	16.9–23.9		3.45	0.06
	benthic	7	22.9 ± 1.2	23.2	20.7–24.0			

*Note*. The table shows the lithostratigraphic unit (TELM), the number of specimens per TELM (*n*), and δ^18^O_p_ mean, median, and range values. Note that tooth specimens analyzed in TELM 2 and 3 include pelagic individuals only, whose statistics are summarized in Table 2. The test indicates no statistical differences between groups with each TELM.

## Data Availability

All data and codes for this study are available on Dryad (Larocca Conte, Aleksinski, et al., 2024).

## Key Points

- Phosphate oxygen isotope values of fossil shark teeth from Seymour Island show no trend across the Eocene
- Consistent, species-specific offsets from bivalve and climate model data indicate movements to warmer, brackish, or colder areas
- Lack of consistent difference between pelagic and benthic sharks suggests that peninsular waters were not strongly stratified

## Plain Language Summary

Sharks have adapted to environmental changes for over 450 million years, but we still do not fully understand how climate change affects them. During the Eocene (56–33.9 million years ago), sharks thrived around the world during warm periods and declined as the planet cooled. Cooling during the Eocene, brought by falling CO_2_ and the opening of tectonic gateways in the Southern Ocean, offers an opportunity to see how sharks experienced climate change and how circumpolar currents formed around Antarctica. Here, we analyze tooth chemistry from pelagic sharks, which live close to the water surface, and benthic sharks, which live close to the seafloor, from Seymour Island off the Antarctic Peninsula, a locality close to the Drake Passage gateway. We use the data to estimate water temperatures experienced by these animals. Surprisingly, benthic sharks do not consistently record the colder water temperatures expected during global cooling and the formation of circumpolar currents near the Drake Passage. Our data suggest that these species moved seasonally to track preferred temperatures as climate cooled. Our study highlights the potential of using shark teeth to investigate habitat use of ancient sharks and to assess how climate change may impact them.
