# Supplementary material for: Eocene Shark Teeth From Peninsular Antarctica: Windows to Habitat Use and Paleoceanography
Source: Paleoceanogr Paleoclimatol. Author manuscript; Available in PMC 2025 Nov 18. (PMC7618383; doi:10.1029/2024PA004965)
Supplement: S17 [file EMS209679-supplement-S17.docx]

Data from: Eocene Shark Teeth from Peninsular Antarctica: Windows to Habitat Use and Paleoceanography.

The repository folder includes scripts and spreadsheets for phosphate oxygen stable isotope (δ^18^O_p_) analysis measured from shark tooth biogenic apatite collected from the Eocene deposits of the La Meseta and Submeseta formations (West Antarctica, Seymour Island). It also contains Fourier-Transform Infrared Spectroscopy (FTIR) analysis, a Bayesian model for temperature estimates, and model output extraction scripts from the iCESM simulation for the Early Eocene (Zhu et al., 2020). Scripts and data are stored in specific folders on the type of analysis. All scripts are in R or Python language.

Usage notes

1 "iCESM modeling scripts" directory

The folder includes scripts in Jupiter Notebook format for extracting and plotting iCESM seawater outputs for the Eocene. The folder includes two files: 1) “d18Ow Analysis Script.ipynb” - This is a Python script primarily using the XArray library, to import iCESM output from Zhu et al. (2020), calculating δ^18^O_w_, and reorganizing the output into monthly time intervals along 25 m and 115 m depth slices, while also averaging output down to these depths; 2) “NetCDF Plotting.ipynb” - this is a Python script primarily using the XArray, Matplotlib, and Cartopy libraries. The script writes a single callable function that creates Matplotlib contour plots from iCESM history output. Variables include temperature, salinity, ideal age, oxygen isotopes, and neodymium isotopes, and map projections include Plate Carree, Mollweide, and orthographic (centering on the Drake Passage). Options are built to enable scale normalization or to set maximum and minimum values for data and select colormaps from a predefined selection of Matplotlib’s “Spectral”, “Viridis”, “Coolwarm”, “GNUplot2”, “PiYG”, “RdYlBu”, and “RdYlGn”. For further questions on model output scripts, please email Adam Aleksinski at [aaleksin@purdue.edu](https://datadryad.org/stash/dataset/doi:10.5061/aaleksin@purdue.edu).

2 "d18O data and maps" directory

The folder includes δ^18^O_p_ of shark tooth bioapatite and other datasets to interpret shark paleoecology. These datasets include:

- δ^18^O_p_ of shark tooth bioapatite (“shark FEST d18Op.csv”). Isotope measurements were run at the Stable Isotope Ecosystem Laboratory of (SIELO) University of California, Merced (California, USA).
- Reference silver phosphate material δ^18^O_p_ for analytical accuracy and precision (“TCEA reference materials.csv). Isotope measurements were run at the Stable Isotope Ecosystem Laboratory of (SIELO) University of California, Merced (California, USA).
- Bulk and serially sampled δ^18^O_c_ data of co-occurring bivalves (Ivany et al., 2008; Judd et al., 2019) (“Ivany et al. 2008_bulk.csv” and “Judd et al., 2019_serial sampling.csv).
- iCESM model temperature and δ^18^O_w_ outputs at 3x and 6x pre-industrial CO_2_ levels for the Early Eocene (Zhu et al., 2020) (“SpinupX3_25m_Mean_Monthly.nc”, “SpinupX6_25m_Mean_Monthly.nc.”, and “CA_x3CO2.csv”). Simulations are integrated from the surface to 25 m.
- δ^18^O values of invertebrate species published in Longinelli (1965) and Longinelli & Nuti (1973), used to convert bulk δ^18^O_c_ (V-SMOW) data of bivalves into δ^18^O_p_ (V-SMOW) values after δ^18^O_c_ (V-PDB) - δ^18^O_c_ (V-SMOW) conversion found in Kim et al. (2015) (“d18O carbonate and phosphate references.csv”).
- R script for data analysis ("d18O data and maps.Rmd”). The script provides annotation through libraries, instrumental accuracy and precision tests, tables, statistical analysis, figures, and model output extractions.

2.1 Dataset description

shark FEST d18Op.csv

- *Sample_ID*: Identification number of tooth specimens.
- *Other_ID*: Temporary identification number of tooth specimens.
- *Taxon*: Species assigned to shark tooth specimens.
- *TELM*: Stratigraphic units of La Meseta (TELM 2-5; ~45 to ~37 Ma) and Submeseta formations (TELMs 6 and 7; ~37 to ~34 Ma) (Amenábar et al., 2020; Douglas et al., 2014; Montes et al., 2013).
- *d18Op*: Mean δ^18^O_p_ values of silver phosphate crystals precipitated from shark tooth bioapatite. Specimens were run in triplicates, corrected, and standardized on the V-SMOW scale.
- *sd*: Standard deviation of silver phosphate triplicate samples per specimen.
- *Protocol*: Silver phosphate protocols used to precipitate crystals from shark tooth bioapatite. We adopted the Rapid UC (“UC_Rapid”) and the SPORA (“SPORA”) protocols after Mine et al. and (2017) Larocca Conte et al. (2024) based on the tooth specimen size and sampling strategy. Descriptions of the methods are included in the main manuscript.
- *Environment*: Inferred shark habitat based on taxonomy classified as benthic or pelagic environment.
- *Collection*: Institutional abbreviations of museum collections from which shark tooth specimens are housed. NRM-PZ is the abbreviation for the Swedish Natural History Museum (Stockholm, Sweden), PRI is the abbreviation for the Paleontological Research Institute (Ithaca, New York, United States), and UCMP is the University of California Museum of Paleontology (Berkeley, California, United States).

TCEA reference materials.csv

- *Identifier_1*: unique identifier number per sample.
- *sample*: reference silver phosphate materials (USGS 80 and USGS 81).
- *amount*: weight of samples in mg.
- *Area 28*: peak area of mass 28 (^12^C^16^O).
- *Area 30*: peak area of mass 30 (^12^C^18^O).
- *d18O_corrected*: corrected δ^18^O_p_ value of reference materials following drift correction, linearity correction, and 2-point calibration to report values on the V-SMOW scale.

Ivany et al. 2008_bulk.csv

- *Telm*: Stratigraphic units of La Meseta (TELM 2-5; ~45 to ~37 Ma) and Submeseta formations (TELMs 6 and 7; ~37 to ~34 Ma) (Amenábar et al., 2020; Douglas et al., 2014; Montes et al., 2013).
- *Locality*: Locality code from which bivalves were collected.
- *Genus*: Genera of bivalves. Specimens are assigned to *Cucullaea* and *Eurhomalea* genera.
- *Line:* Sampling areas of specimens. The sampling strategy is described in Ivany et al. (2008).
- *d13C*: δ^13^C values of specimens from sampled lines. Values are reported in the V-PDB scale.
- *d18Oc_PDB*: δ^18^O_c_ values of specimens from sampled lines. Values are reported in the V-PDB scale.

Judd et al., 2019_serial sampling.csv

- *Horizon:* horizons of the TELM 5 unit (La Meseta Formation) from which bivalves were collected. Horizon 1 is stratigraphically the lowest, while horizon 4 is the highest (Judd et al., 2019).
- *ID*: Identification number of specimens.
- *Latitude*: Geographic coordinate where bivalve specimens were collected.
- *Longitude*: Geographic coordinate where bivalve specimens were collected.
- *Surface sampled*: Specific sampling area, indicating whether sampling occurred in the interior or exterior portion of shells.
- *distance*: The distance from the umbo in mm from which sampling occurred along a single shell.
- *d18Oc_PDB*: δ^18^O_c_ values of specimens from sampled areas of shells. Values are reported on the V-PDB scale.

SpinupX3_25m_Mean_Monthly.nc

See section 1 ("iCESM modeling scripts" directory, “d18Ow Analysis Script.ipynb” script) for a full description of the iCESM model output extraction.

SpinupX6_25m_Mean_Monthly.nc

See section 1 ("iCESM modeling scripts" directory, “d18Ow Analysis Script.ipynb” script) for a full description of the iCESM model output extraction.

CA_x3CO2.csv

- *lat*: Geographic coordinate where temperature and δ^18^O_w_ model values are extracted from the iCESM simulation scaled at 3x preindustrial CO_2_ levels (values averaged within a seawater column depth of 25 m).
- *long*: Geographic coordinate where temperature and δ^18^O_w_ model values are extracted from the iCESM simulation scaled at 3x preindustrial CO_2_ levels (values averaged within a seawater column depth of 25 m).
- *T_mean*: Simulated seawater temperature values in °C.
- *d18Ow*: Simulated seawater δ^18^O_w_ values (V-SMOW).
- *d18Op*: Simulated seawater δ^18^O_p_ values (V-SMOW). Values were calculated by using seawater temperature and δ^18^O_w_ arrays following the paleothermometer equation after Lécuyer et al. (2013).

d18O carbonate and phosphate references.csv

- *species*: Species of invertebrate taxa.
- *type*: Specimen type, including barnacles, brachiopods, crabs, and mollusks.
- *depth*: Depth of seawater column where specimens were collected, reported in meters below sea level when specified.
- *d18Op*: δ^18^O_p_ values of invertebrate specimens (V-SMOW).
- *d18Oc_PDB*: δ^18^O_c_ values of invertebrate specimens (V-PDB).
- *Reference*: Citations from which data were taken to build the dataset (Longinelli, 1965; Longinelli & Nuti, 1973).

TELM diversity.csv

- *genus:* genera of sharks and rays compiled from literature (Engelbrecht et al., 2016a, 2016b, 2017a, 2017b, 2019; Kriwet, 2005; Kriwet et al., 2016; Long, 1992; Marramá et al., 2018).
- *species*: species of sharks and rays compiled from literature (Engelbrecht et al., 2016a, 2016b, 2017a, 2017b, 2019; Kriwet, 2005; Kriwet et al., 2016; Long, 1992; Marramá et al., 2018).
- *Environment*: Inferred shark habitat based on taxonomy classified as benthic or pelagic environment.
- *TELM:* Stratigraphic units of La Meseta (TELM 1-5; ~44 to ~37 Ma) and Submeseta formations (TELMs 6 and 7; ~37 to ~34 Ma) (Amenábar et al., 2020; Douglas et al., 2014; Montes et al., 2013).

3 “FTIR data” directory

The folder includes FTIR acquisitions and data analysis scripts on reference materials and shark tooth bioapatite for quality checks to test diagenesis effects on δ^18^O_p_ of sharks. The folder includes:

- The R project file “apatite_ftir.Rproj”. This project file navigates through scripts for raw data processing and data analysis. The background of the raw data was processed following custom R functions from Trayler et al. (2023; <https://github.com/robintrayler/collagen_demineralization>).
- The “.Rproj.user” folder includes project-specific temporary files (e.g. auto-saved source documents, window-state, etc.) stored by the R project file “apatite_ftir.Rproj”. The folder may be hidden depending on directory view options.
- The “raw data” directory stores spectra acquisitions as .dpt files. Spectra files are stored in the folders “apatite” and “calcite” based on the material type. Spectra were obtained in the 400 – 4000 cm⁻¹ range using a Bruker Vertex 70 Far-Infrared in ATR located at the Nuclear Magnetic Resonance Facility at the University of California Merced (California, USA).
- The “processed” directory includes processed spectra stored as .csv files (“apatite_data.csv” and “calcite_data.csv”) following the background correction (Trayler et al., 2023) and processed infrared data from Larocca Conte et al. (2024) (“Larocca Conte et al._SPORA_apatite_data.csv”) from which the NIST SRM 120c spectrum was filtered. Infrared spectra data in “Larocca Conte et al._SPORA_apatite_data.csv” were obtained and corrected following the same methodologies mentioned above.
- The “R” directory includes R scripts of customized source functions for background correction (Trayler et al., 2023; inspect the "functions" directory and the R script "0_process_data.R") and data analysis (“data_analysis.R”). The scripts provide annotation through libraries and functions used for data processing and analysis.
- Additional datasets. The “data_FTIR_d18O.csv” includes infrared data and δ^18^O_p_ values of specimens, while the “Grunenwald et al., 2014_CO3.csv” is the dataset after Grunenwald et al. (2014) used to predict carbonate content from the materials featured in this work.

3.1 Dataset description

Spreadsheets included in the “processed” directory

The datasets “apatite_data.csv”, “calcite_data.csv”, and “Larocca Conte et al._SPORA_apatite_data.csv” are structured with the following variables:

- *wavenumber*: infrared wavenumber in cm^-1^.
- *absorbance*: infrared absorbance value.
- *file_name:* .dpt file name from which infrared wavenumber and absorbance values were obtained following the background correction.

data_FTIR_d18O.csv

- *file_name:* .dpt file name from which infrared wavenumber and absorbance values were obtained following the background correction.
- *v4PO4_565_wavenumber*: Wavenumber of maximum infrared absorbance around the first νPO_4_ band, usually at 565 cm^-1^.
- *v4PO4_565*: Peak absorbance value of the first ν_4_PO_4_ band (~565 cm^-1^).
- *v4PO4_valley_wavenumber*: Wavenumber of valley between ν_4_PO_4_ bands.
- *v4PO4_valley*: Absorbance value of the valley between ν_4_PO_4_ bands.
- *v4PO4_603_wavenumber*: Wavenumber of maximum infrared absorbance around the second ν_4_PO_4_ band, usually at 603 cm^-1^.
- *v4PO4_603*: Peak absorbance value of the second ν_4_PO_4_ band (~603 cm^-1^).
- *CI*: Crystallinity index calculated after equation provided in (Shemesh, 1990) as (*v4PO4_565* + *v4PO4_603* / *v4PO4_valley*) (i.e., the sum of peak absorbance of νPO4 bands divided by the absorbance value of the valley between peaks).
- *material*: Material type of samples (i.e., standard material, enameloid, dentin sampled from the crown or root area of shark teeth, and enameloid mixed with dentin).
- *AUC_v3PO4*: Area under the curve of the ν_3_PO_4_ and ν_1_PO_4_ bands where maximum absorbance is at ~1025 cm^-1^ and ~960 cm^-1^, respectively.
- *AUC_v3CO3*: Area under the curves of Type-A and Type-B carbonate bands having maximum infrared absorbance at ~1410 (Type-B), ~1456 (Type-B), and ~1545 cm^-1^ (Type-A).
- *v3CO3_v3PO4_ratio*: Ratio between area under the curves of carbonate and phosphate bands (i.e., *AUC_v3CO3* / *AUC_v3PO4*).
- *CO3_wt*: Estimated mean carbonate content following the equation in Grunenwald et al. (2014) (i.e. *CO3_wt* = 28.4793 (±1.4803) * *v3CO3_v3PO4_ratio* + 0.1808(±0.2710); R^2^ = 0.985).
- *CO3_wt_sd*: Standard deviation of estimated carbonate content calculated by propagating the error around coefficients provided in the Grunenwald et al. (2014) equation (see full equation in *CO3_wt*).
- *Taxon*: Species assigned to shark tooth specimens.
- *TELM*: Stratigraphic units of La Meseta (TELM 2-5; ~45 to ~37 Ma) and Submeseta formations (TELMs 6 and 7; ~37 to ~34 Ma) (Amenábar et al., 2020; Douglas et al., 2014; Montes et al., 2013).
- *d18Op*: Mean δ^18^O_p_ values of silver phosphate crystals precipitated from shark tooth bioapatite. Specimens were run in triplicates, corrected, and standardized on the V-SMOW scale.
- *sd*: Standard deviation of silver phosphate triplicate samples per specimen.
- *Collection*: Institutional abbreviations of museum collections where shark tooth specimens are housed. Infrared spectra were obtained from a selected subset of tooth specimens in the care of the Swedish Natural History Museum (NRM-PZ; Stockholm, Sweden).

Grunenwald et al., 2014_CO3.csv

- *sample*: Sample code.
- *material*: Material type of samples (i.e., standard material, bone, and enamel).
- *v3CO3*: Area under the curves of Type-A and Type-B carbonate bands having maximum infrared absorbance at ~1410 (Type-B), ~1456 (Type-B), and ~1545 cm-1 (Type-A).
- *v3PO4*: *AUC_v3PO4*: Area under the curve of the ν_3_PO_4_ and ν_1_PO_4_ bands where maximum absorbance is at ~1025 cm^-1^ and ~960 cm^-1^, respectively.
- *v3CO3_v3PO4_ratio*: *v3CO3_v3PO4_ratio*: Ratio between area under the curves of carbonate and phosphate bands (i.e., *v3CO3* / *v3PO4*).
- *CO3_wt*: Carbonate content measured via CO_2_ coulometry. Further details about the analytical measurements are found in Grunenwald et al. (2014).

4 “Bayes_FEST_Temperautre Estimates” directory

The folder includes the Bayesian approach used to estimate posterior seawater temperature, δ^18^O_w_ values from δ^18^O_p_ of sharks bioapatite using a Bayesian approach modified after Griffiths et al. (2023). The original scripts used in Griffiths et al. (2023) are reposited here: <https://github.com/robintrayler/bayesian_phosphate>. The directory includes:

- The R project file “Bayes_FEST.Rproj”. This project file navigates through scripts for raw data analysis.
- The “.Rproj.user” folder includes project-specific temporary files (e.g. auto-saved source documents, window-state, etc.) stored by the R project file “Bayes_FEST.Rproj”. The folder may be hidden depending on directory view options.
- The “data” folder includes the spreadsheets for modeled seawater temperature and δ^18^O_w_ values (“CA_x3CO2.csv”) and δ^18^O_p_ values of shark tooth bioapatite (“shark FEST d18Op.csv”) used as prior information for the Bayesian model. We refer to section 2.1 for the full description of spreadsheets.
- The “R” folder includes customized functions for the Bayesian model stored in the “functions” directory and the script for data analysis (“01_model_sharks.R”). The script includes a comparison of paleothermometer equations after Kolodny et al. (1983), Lécuyer et al. (2013), Longinelli & Nuti (1973), and (Pucéat et al. (2010) using the bulk δ^18^O_p_ shark tooth bioapatite, simulated seawater temperature and δ^18^O_w_ values as prior inputs. While all paleothermometers estimate similar posterior bulk δ^18^O_p_ close to empirical values, temperature estimates using the Pucéat et al. (2010) method are often the highest, generating estimates ~8°C higher than other equations. We therefore used the Lécuyer et al. (2013) paleothermomether for temperature estimates using δ^18^O_p_ of shark bioapatite grouped by taxa because it:

1. Provides consistent posterior temperature estimates relative to other equations (Longinelli & Nuti, 1973, Kolodny et al., 1983).
2. provides temperature values from fish tooth specimens consistent with estimates of co-existing bivalves or brachiopod carbonate shells.

The script provides annotation through libraries, statistical analysis, figures, and tables.

4 Software

4.1 R

R and R Studio (R Development Core Team, 2024; RStudio Team, 2024) are required to run scripts included in the "d18O data and maps", “FTIR data”, and “Bayes_FEST_Temperautre Estimates” directories, which were created using versions 4.4.1 and 2024.04.02, respectively. Install the following libraries before running scripts:

“cowplot” (Wilke, 2024), “colorspace” (Zeileis et al., 2020), “DescTools” (Signorell, 2024), “lattice” (Sarkar, 2008), “flextable” (Gohel & Skintzos, 2024), “ggh4x” (van den Brand, 2024), “ggnewscale” (Campitelli, 2024), “ggpubr” (Kassambara, 2023a), “ggspatial” (Dunnington, 2023), “ggstance” (Henry et al., 2024), “ggstar” (Xu, 2022), “greekLetters” (Kévin Allan Sales Rodrigues, 2023), “gridExtra” (Auguie, 2017), “mapdata” (code by Richard A. Becker & version by Ray Brownrigg., 2022); “mapproj” (for R by Ray Brownrigg et al., 2023), “maps” (code by Richard A. Becker et al., 2023), “ncdf4” (Pierce, 2023), “oce” (Kelley & Richards, 2023), “rasterVis” (Oscar Perpiñán & Robert Hijmans, 2023), “RColorBrewer” (Neuwirth, 2022), “rnaturalearth” (Massicotte & South, 2023), “rnaturalearthhires” (South et al., 2024),”rstatix” (Kassambara, 2023b), “scales” (Wickham et al., 2023), “tidyverse” (Wickham et al., 2019), “viridisLite” (Garnier et al., 2023).

4.2 Python

Python scripts, including “d18O Analysis Script.ipynb” and “NetCDF Plotting.ipynb”, utilize the Jupyter Notebook interactive ‘platform and are executed using Python version 3.9.16. Install the following libraries before running scripts:

“xarray” (Hoyer & Joseph, 2017), “matplotlib” (Hunter, 2007), “cartopy” (Met Office, 2015).

5 References

Amenábar, C. R., Montes, M., Nozal, F., & Santillana, S. (2020). Dinoflagellate cysts of the la Meseta Formation (middle to late Eocene), Antarctic Peninsula: Implications for biostratigraphy, palaeoceanography and palaeoenvironment. *Geological Magazine*, *157*(3), 351–366. https://doi.org/10.1017/S0016756819000591

Auguie, B. (2017). gridExtra: Miscellaneous Functions for “Grid” Graphics. Retrieved from https://cran.r-project.org/package=gridExtra

van den Brand, T. (2024). ggh4x: Hacks for “ggplot2.” Retrieved from https://cran.r-project.org/package=ggh4x

Campitelli, E. (2024). ggnewscale: Multiple Fill and Colour Scales in “ggplot2.” Retrieved from https://cran.r-project.org/package=ggnewscale

code by Richard A. Becker, O. S., & version by Ray Brownrigg., A. R. W. R. (2022). mapdata: Extra Map Databases. Retrieved from https://cran.r-project.org/package=mapdata

code by Richard A. Becker, O. S., version by Ray Brownrigg. Enhancements by Thomas P Minka, A. R. W. R., & Deckmyn., A. (2023). maps: Draw Geographical Maps. Retrieved from https://cran.r-project.org/package=maps

Douglas, P. M. J., Affek, H. P., Ivany, L. C., Houben, A. J. P., Sijp, W. P., Sluijs, A., et al. (2014). Pronounced zonal heterogeneity in Eocene southern high-latitude sea surface temperatures. *Proceedings of the National Academy of Sciences of the United States of America*, *111*(18), 6582–6587. https://doi.org/10.1073/pnas.1321441111

Dunnington, D. (2023). ggspatial: Spatial Data Framework for ggplot2. Retrieved from https://cran.r-project.org/package=ggspatial

Engelbrecht, A., Mörs, T., Reguero, M. A., & Kriwet, J. (2016a). A new sawshark, Pristiophorus laevis, from the Eocene of Antarctica with comments on Pristiophorus lanceolatus. *Historical Biology*, *29*(6), 841–853. https://doi.org/10.1080/08912963.2016.1252761

Engelbrecht, A., Mörs, T., Reguero, M. A., & Kriwet, J. (2016b). Revision of Eocene Antarctic carpet sharks (Elasmobranchii, Orectolobiformes) from Seymour Island, Antarctic Peninsula. *Journal of Systematic Palaeontology*, *15*(12), 969–990. https://doi.org/10.1080/14772019.2016.1266048

Engelbrecht, A., Mörs, T., Reguero, M. A., & Kriwet, J. (2017a). Eocene squalomorph sharks (Chondrichthyes, Elasmobranchii) from Antarctica. *Journal of South American Earth Sciences*, *78*, 175–189. https://doi.org/10.1016/j.jsames.2017.07.006

Engelbrecht, A., Mörs, T., Reguero, M. A., & Kriwet, J. (2017b). New carcharhiniform sharks (Chondrichthyes, Elasmobranchii) from the early to middle Eocene of Seymour Island, Antarctic Peninsula. *Journal of Vertebrate Paleontology*, *37*(6). https://doi.org/10.1080/02724634.2017.1371724

Engelbrecht, A., Mörs, T., Reguero, M. A., & Kriwet, J. (2019). Skates and rays (Elasmobranchii, Batomorphii) from the Eocene La Meseta and Submeseta formations, Seymour Island, Antarctica. *Historical Biology*, *31*(8), 1028–1044. https://doi.org/10.1080/08912963.2017.1417403

for R by Ray Brownrigg, D. M. P., Minka, T. P., & transition to Plan 9 codebase by Roger Bivand. (2023). mapproj: Map Projections. Retrieved from https://cran.r-project.org/package=mapproj

Garnier, Simon, Ross, Noam, Rudis, Robert, et al. (2023). {viridis(Lite)} - Colorblind-Friendly Color Maps for R. https://doi.org/10.5281/zenodo.4678327

Gohel, D., & Skintzos, P. (2024). flextable: Functions for Tabular Reporting. Retrieved from https://cran.r-project.org/package=flextable

Griffiths, M. L., Eagle, R. A., Kim, S. L., Flores, R. J., Becker, M. A., IV, H. M. M., et al. (2023). Endothermic physiology of extinct megatooth sharks. *Proceedings of the National Academy of Sciences*, *120*(27), e2218153120. https://doi.org/10.1073/PNAS.2218153120

Grunenwald, A., Keyser, C., Sautereau, A. M., Crubézy, E., Ludes, B., & Drouet, C. (2014). Revisiting carbonate quantification in apatite (bio)minerals: A validated FTIR methodology. *Journal of Archaeological Science*, *49*(1), 134–141. https://doi.org/10.1016/j.jas.2014.05.004

Henry, L., Wickham, H., & Chang, W. (2024). ggstance: Horizontal “ggplot2” Components. Retrieved from https://cran.r-project.org/package=ggstance

Hoyer, S., & Joseph, H. (2017). xarray: N-D labeled Arrays and Datasets in Python. *Journal of Open Research Software*, *5*(1), 17. https://doi.org/10.5334/jors.148

Hunter, J. D. (2007). Matplotlib: A 2D graphics environment. *Computing in Science & Engineering*, *9*(3), 90–95. https://doi.org/10.1109/MCSE.2007.55

Ivany, L. C., Lohmann, K. C., Hasiuk, F., Blake, D. B., Glass, A., Aronson, R. B., & Moody, R. M. (2008). Eocene climate record of a high southern latitude continental shelf: Seymour Island, Antarctica. *Bulletin of the Geological Society of America*, *120*(5–6), 659–678. https://doi.org/10.1130/B26269.1

Judd, E. J., Ivany, L. C., DeConto, R. M., Halberstadt, A. R. W., Miklus, N. M., Junium, C. K., & Uveges, B. T. (2019). Seasonally Resolved Proxy Data From the Antarctic Peninsula Support a Heterogeneous Middle Eocene Southern Ocean. *Paleoceanography and Paleoclimatology*, *34*(5), 787–799. https://doi.org/10.1029/2019PA003581

Kassambara, A. (2023a). ggpubr: “ggplot2” Based Publication Ready Plots. Retrieved from https://cran.r-project.org/package=ggpubr

Kassambara, A. (2023b). rstatix: Pipe-Friendly Framework for Basic Statistical Tests. Retrieved from https://cran.r-project.org/package=rstatix

Kelley, D., & Richards, C. (2023). oce: Analysis of Oceanographic Data. Retrieved from https://cran.r-project.org/package=oce

Kévin Allan Sales Rodrigues. (2023). greekLetters: routines for writing Greek letters and mathematical symbols on the RStudio and RGui. Retrieved from https://cran.r-project.org/package=greekLetters

Kolodny, Y., Luz, B., & Navon, O. (1983). Oxygen isotope variations in phosphate of biogenic apatites, I. Fish bone apatite-rechecking the rules of the game. *Earth and Planetary Science Letters*, *64*(3), 398–404. https://doi.org/10.1016/0012-821X(83)90100-0

Kriwet, J. (2005). Additions to the Eocene selachian fauna of Antarctica with comments on Antarctic selachian diversity. *Journal of Vertebrate Paleontology*, *25*(1), 1–7. https://doi.org/10.1671/0272-4634(2005)025[0001:ATTESF]2.0.CO;2

Kriwet, J., Engelbrecht, A., Mörs, T., Reguero, M., & Pfaff, C. (2016). Ultimate Eocene (Priabonian) chondrichthyans (Holocephali, Elasmobranchii) of Antarctica. *Journal of Vertebrate Paleontology*, *36*(4). https://doi.org/10.1080/02724634.2016.1160911

Larocca Conte, G., Lopes, L. E., Mine, A. H., Trayler, R. B., & Kim, S. L. (2024). SPORA, a new silver phosphate precipitation protocol for oxygen isotope analysis of small, organic-rich bioapatite samples. *Chemical Geology*, *651*, 122000. https://doi.org/10.1016/J.CHEMGEO.2024.122000

Lécuyer, C., Amiot, R., Touzeau, A., & Trotter, J. (2013). Calibration of the phosphate δ18O thermometer with carbonate-water oxygen isotope fractionation equations. *Chemical Geology*, *347*, 217–226. https://doi.org/10.1016/j.chemgeo.2013.03.008

Long, D. J. (1992). Sharks from the La Meseta Formation (Eocene), Seymour Island, Antarctic Peninsula. *Journal of Vertebrate Paleontology*, *12*(1), 11–32. https://doi.org/10.1080/02724634.1992.10011428

Longinelli, A. (1965). Oxygen isotopic composition of orthophosphate from shells of living marine organisms. *Nature*, *207*(4998), 716–719. https://doi.org/10.1038/207716a0

Longinelli, A., & Nuti, S. (1973). Revised phosphate-water isotopic temperature scale. *Earth and Planetary Science Letters*, *19*(3), 373–376. https://doi.org/10.1016/0012-821X(73)90088-5

Marramá, G., Engelbrecht, A., Mörs, T., Reguero, M. A., & Kriwet, J. (2018). The southernmost occurrence of Brachycarcharias (Lamniformes, Odontaspididae) from the Eocene of Antarctica provides new information about the paleobiogeography and paleobiology of Paleogene sand tiger sharks. *Rivista Italiana Di Paleontologia e Stratigrafia*, *124*(2), 283–297.

Massicotte, P., & South, A. (2023). rnaturalearth: World Map Data from Natural Earth. Retrieved from https://cran.r-project.org/package=rnaturalearth

Met Office. (2015). Cartopy: a cartographic python library with a Matplotlib interface. Exeter, Devon. Retrieved from https://scitools.org.uk/cartopy

Mine, A. H., Waldeck, A., Olack, G., Hoerner, M. E., Alex, S., & Colman, A. S. (2017). Microprecipitation and δ18O analysis of phosphate for paleoclimate and biogeochemistry research. *Chemical Geology*, *460*(March), 1–14. https://doi.org/10.1016/j.chemgeo.2017.03.032

Montes, M., Nozal, F., Santillana, S., Marenssi, S., & Olivero, E. (2013). Mapa Geológico de Isla Marambio (Seymour), Antártida, escala 1:20,000. *Serie Cartográfica*.

Neuwirth, E. (2022). RColorBrewer: ColorBrewer Palettes. Retrieved from https://cran.r-project.org/package=RColorBrewer

Oscar Perpiñán, & Robert Hijmans. (2023). rasterVis. Retrieved from https://oscarperpinan.github.io/rastervis/

Pierce, D. (2023). ncdf4: Interface to Unidata netCDF (Version 4 or Earlier) Format Data Files. Retrieved from https://cran.r-project.org/package=ncdf4

Pucéat, E., Joachimski, M. M., Bouilloux, A., Monna, F., Bonin, A., Motreuil, S., et al. (2010). Revised phosphate-water fractionation equation reassessing paleotemperatures derived from biogenic apatite. *Earth and Planetary Science Letters*, *298*(1–2), 135–142. https://doi.org/10.1016/j.epsl.2010.07.034

R Development Core Team. (2024). A language and environment for statistical computing. R Foundation for Statistical Computing, Vienna, Austria. Vienna, Austria.

RStudio Team. (2024). RStudio: Integrated Development for R. Boston, MA: RStudio, PBC. Retrieved from http://www.rstudio.com/.

Sarkar, D. (2008). *Lattice: Multivariate Data Visualization with R*. New York: Springer. Retrieved from http://lmdvr.r-forge.r-project.org

Shemesh, A. (1990). Crystallinity and diagenesis of sedimentary apatites. *Geochimica et Cosmochimica Acta*, *54*(9), 2433–2438. https://doi.org/10.1016/0016-7037(90)90230-I

Signorell, A. (2024). DescTools: Tools for Descriptive Statistics. Retrieved from https://cran.r-project.org/package=DescTools

South, A., Michael, S., & Massicotte, P. (2024). rnaturalearthhires: High Resolution World Vector Map Data from Natural Earth used in rnaturalearth. Retrieved from https://github.com/ropensci/rnaturalearthhires

Trayler, R. B., Landa, P. V., & Kim, S. L. (2023). Evaluating the efficacy of collagen isolation using stable isotope analysis and infrared spectroscopy. *Journal of Archaeological Science*, *151*, 105727. https://doi.org/10.1016/j.jas.2023.105727

Wickham, H., Averick, M., Bryan, J., Chang, W., McGowan, L. D., François, R., et al. (2019). Welcome to the {tidyverse}. *Journal of Open Source Software*, *4*(43), 1686. https://doi.org/10.21105/joss.01686

Wickham, H., Pedersen, T. L., & Seidel, D. (2023). scales: Scale Functions for Visualization. Retrieved from https://cran.r-project.org/package=scales

Wilke, C. O. (2024). cowplot: Streamlined Plot Theme and Plot Annotations for “ggplot2.” Retrieved from https://cran.r-project.org/package=cowplot

Xu, S. (2022). ggstar: Multiple Geometric Shape Point Layer for “ggplot2.” Retrieved from https://cran.r-project.org/package=ggstar

Zeileis, A., Fisher, J. C., Hornik, K., Ihaka, R., McWhite, C. D., Murrell, P., et al. (2020). {colorspace}: A Toolbox for Manipulating and Assessing Colors and Palettes. *Journal of Statistical Software*, *96*(1), 1–49. https://doi.org/10.18637/jss.v096.i01

Zhu, J., Poulsen, C. J., Otto-Bliesner, B. L., Liu, Z., Brady, E. C., & Noone, D. C. (2020). Simulation of early Eocene water isotopes using an Earth system model and its implication for past climate reconstruction. *Earth and Planetary Science Letters*, *537*, 116164. https://doi.org/10.1016/j.epsl.2020.116164
