# Supplementary material for: Eocene Shark Teeth From Peninsular Antarctica: Windows to Habitat Use and Paleoceanography
Source: Paleoceanogr Paleoclimatol. Author manuscript; Available in PMC 2025 Nov 18. (PMC7618383; doi:10.1029/2024PA004965)
Supplement: Supporting Information [file EMS209679-supplement-Supporting_Information.pdf]

References From the Supporting Information

Al Sekhaneh, W., Akkam, Y. H., Kamel, G., Drabee, A., & Popp, J. (2021). Investigation of ancient teeth using Raman spectroscopy and synchrotron radiation Fourier-transform infrared (SR-μFTIR): Mapping and novel method of dating. *Digest Journal of Nanomaterials and Biostructures*, *16*(2), 713–724. https://doi.org/10.15251/djnb.2021.162.713

Amir, S., Hafidi, M., Merlina, G., Hamdi, H., & Revel, J.-C. (2004). Elemental analysis, FTIR and 13C-NMR of humic acids from sewage sludge composting. *Agronomie*, *24*(1), 13–18. https://doi.org/10.1051/agro

Aoba, T., Miake, Y., Shimoda, S., Prostak, K., Moreno, E. C., & Suga, S. (1991). Dental apatites in in vertebrate species: Morphology and chemical properties BT. In S. Suga, & H. Nakahara (Eds.), *Mechanisms and phylogeny of mineralization in biological systems* (pp. 459–463). Springer Japan.

de Lopes, C. C. A., Limirio, P. H. J. O., Novais, V. R., & Dechichi, P. (2018). Fourier transform infrared spectroscopy (FTIR) application chemical characterization of enamel, dentin and bone. *Applied Spectroscopy Reviews*, *53*(9), 747–769. https://doi.org/10.1080/05704928.2018.1431923

Dorozhkin, S. V. (1997). Surface reactions of apatite dissolution. *Journal of Colloid and Interface Science*, *191*(2), 489–497. https://doi.org/10.1006/jcis.1997.4942

Enax, J., Janus, A. M., Raabe, D., Epple, M., & Fabritius, H. O. (2014). Ultrastructural organization and micromechanical properties of shark tooth enameloid. In *Acta biomaterialia*, (Vol. 10, pp. 3959–3968). Elsevier Ltd. https://doi.org/10.1016/j.actbio.2014.04.028

Grimes, V., & Pellegrini, M. (2013). A comparison of pretreatment methods for the analysis of phosphate oxygen isotope ratios in bioapatite. *Rapid Communications in Mass Spectrometry*, *27*(3), 375–390. https://doi.org/10.1002/rcm.6463

Grunenwald, A., Keyser, C., Sautereau, A. M., Crubézy, E., Ludes, B., & Drouet, C. (2014a). Novel contribution on the diagenetic physicochemical features of bone and teeth minerals, as substrates for ancient DNA typing. *Analytical and Bioanalytical Chemistry*, *406*(19), 4691–4704. https://doi.org/10.1007/s00216-014-7863-z

Grunenwald, A., Keyser, C., Sautereau, A. M., Crubézy, E., Ludes, B., & Drouet, C. (2014b). Revisiting carbonate quantification in apatite (bio) minerals: A validated FTIR methodology. *Journal of Archaeological Science*, *49*(1), 134–141. https://doi.org/10.1016/j.jas.2014.05.004

Koch, P. L., Tuross, N., & Fogel, M. L. (1997). The effects of sample treatment and diagenesis on the isotopic integrity of carbonate in biogenic hydroxylapatite. *Journal of Archaeological Science*, *24*(5), 417–429. https://doi.org/10.1006/jasc.1996.0126

Lebon, M., Reiche, I., Gallet, X., Bellot-Gurlet, L., & Zazzo, A. (2016). Rapid quantification of bone collagen content by ATR-FTIR spectroscopy. *Radiocarbon*, *58*(1), 131–145. https://doi.org/10.1017/RDC.2015.11

Lécuyer, C. (2004). Oxygen isotope analysis of phosphate. In *Handbook of stable isotope analytical techniques* (pp. 482–496). https://doi.org/10.1016/B978-044451114-0/50024-7

Lécuyer, C., Balter, V., Martineau, F., Fourel, F., Bernard, A., Amiot, R., et al. (2010). Oxygen isotope fractionation between apatite-bound carbonate and water determined from controlled experiments with synthetic apatites precipitated at 10-37°C. *Geochimica et Cosmochimica Acta*, *74*(7), 2072–2081. https://doi.org/10.1016/j.gca.2009.12.024

Lee-Thorp, J. (2002). Two decades of progress towards understanding fossilization processes and isotopic signals in calcified tissue minerals. *Archaeometry*, *44*(3), 435–446. https://doi.org/10.1111/1475-4754.t01-1-00076

LeGeros, R. Z. (1981). Apatites in biological systems. *Progress in Crystal Growth and Characterization*, *4*(1–2), 1–45. https://doi.org/10.1016/0146-3535(81)90046-0

LeGeros, R. Z., Bonel, G., & Legros, R. (1978). Types of "H_2O" in human enamel and in precipitated apatites. *Calcified Tissue Research*, *26*(1), 111–118. https://doi.org/10.1007/BF02013245/METRICS

Leventouri, T. (2006). Synthetic and biological hydroxyapatites: Crystal structure questions. *Biomaterials*, *27*(18), 3339–3342. https://doi.org/10.1016/j.biomaterials.2006.02.021

Longinelli, A., & Nuti, S. (1973). Revised phosphate-water isotopic temperature scale. *Earth and Planetary Science Letters*, *19*(3), 373–376. https://doi.org/10.1016/0012-821X(73)90088-5

Lübke, A., Enax, J., Loza, K., Prymak, O., Gaengler, P., Fabritius, H. O., et al. (2015). Dental lessons from past to present: Ultrastructure and composition of teeth from plesiosaurs, dinosaurs, extinct and recent sharks. *RSC Advances*, *5*(76), 61612–61622. https://doi.org/10.1039/c5ra11560d

Miake, Y., Aoba, T., Moreno, E. C., Shimoda, S., Prostak, K., & Suga, S. (1991). Ultrastructural studies on crystal growth of enameloid minerals in elasmobranch and teleost fish. *Calcified Tissue International*, *48*(3), 204–217. https://doi.org/10.1007/BF02570556

Moreno, E. C., & Aoba, T. (1991). S. Suga, & H. Nakahara (Eds.), *Formation and solubility of carbonated tooth minerals BT—Mechanisms and phylogeny of mineralization in biological systems* (pp. 179–186). Springer Japan.

National Institute of Standards and Technology. (1988). Standard reference material 120c Florida phosphate rock.

Pederzani, S., Snoeck, C., Wacker, U., & Britton, K. (2020). Anion exchange resin and slow precipitation preclude the need for pretreatments in silver phosphate preparation for oxygen isotope analysis of bioapatites. *Chemical Geology*, *534*, 119455. https://doi.org/10.1016/j.chemgeo.2019.119455

Pucéat, E., Reynard, B., & Lécuyer, C. (2004). Can crystallinity be used to determine the degree of chemical alteration of biogenic apatites? *Chemical Geology*, *205*(1–2), 83–97. https://doi.org/10.1016/j.chemgeo.2003.12.014

Ren, F., Ding, Y., & Leng, Y. (2014). Infrared spectroscopic characterization of carbonated apatite: A combined experimental and computational study. *Journal of Biomedical Materials Research—Part A*, *102*(2), 496–505. https://doi.org/10.1002/jbm.a.34720

Roche, D., Ségalen, L., Balan, E., & Delattre, S. (2010). Preservation assessment of Miocene-Pliocene tooth enamel from Tugen Hills (Kenyan Rift Valley) through FTIR, chemical and stable-isotope analyses. *Journal of Archaeological Science*, *37*(7), 1690–1699. https://doi.org/10.1016/j.jas.2010.01.029

Shemesh, A. (1990). Crystallinity and diagenesis of sedimentary apatites. *Geochimica et Cosmochimica Acta*, *54*(9), 2433–2438. https://doi.org/10.1016/0016-7037(90)90230-I

Souza, K. K. D., Schaefer, C. E. G. R., Simas, F. N. B., Spinola, D. N., & de Paula, M. D. (2014). Soil formation in Seymour Island, Weddell Sea, Antarctica. *Geomorphology*, *225*(C), 87–99. https://doi.org/10.1016/j.geomorph.2014.03.047

Trueman, C. N., Privat, K., & Field, J. (2008). Why do crystallinity values fail to predict the extent of diagenetic alteration of bone mineral? *Palaeogeography, Palaeoclimatology, Palaeoecology*, *266*(3–4), 160–167. https://doi.org/10.1016/j.palaeo.2008.03.038

Wiedemann-Bidlack, F. B., Colman, A. S., & Fogel, M. L. (2008). Phosphate oxygen isotope analysis on microsamples of bioapatite: Removal of organic contamination and minimization of sample size. *Rapid Communications in Mass Spectrometry*, *22*(12), 1457–1466. https://doi.org/10.1002/rcm.3553
